# ﻿Refining the phylogeny and taxonomy of the apple tribe Maleae (Rosaceae): insights from phylogenomic analyses of 563 plastomes and a taxonomic synopsis of *Photinia* and its allies in the Old World

**DOI:** 10.3897/phytokeys.242.117481

**Published:** 2024-05-31

**Authors:** Hui Wang, Xiao-Ya Li, Yan Jiang, Ze-Tao Jin, Dai-Kun Ma, Bing Liu, Chao Xu, Bin-Jie Ge, Ting Wang, Qiang Fan, Shui-Hu Jin, Guang-Ning Liu, Bin-Bin Liu

**Affiliations:** 1 College of Forestry and Biotechnology, Zhejiang Agriculture and Forestry University, Hangzhou, Zhejiang 311300, China Zhejiang Agriculture and Forestry University Hangzhou China; 2 State Key Laboratory of Plant Diversity and Specialty Crops, Institute of Botany, Chinese Academy of Sciences, Beijing 100093, China Institute of Botany, Chinese Academy of Sciences Beijing China; 3 China National Botanical Garden, Beijing 100093, China China National Botanical Garden Beijing China; 4 University of Chinese Academy of Sciences, Beijing 100049, China University of Chinese Academy of Sciences Beijing China; 5 Key Laboratory of Plant Resources Conservation and Sustainable Utilization, South China Botanical Garden, Chinese Academy of Sciences, Guangzhou, Guangdong 510650, China South China Botanical Garden, Chinese Academy of Sciences Guangzhou China; 6 College of Horticulture, State Key Laboratory of Crop Genetics & Germplasm Enhancement and Utilization, Nanjing Agricultural University, Nanjing, Jiangsu 210095, China Nanjing Agricultural University Nanjing China; 7 Eastern China Conservation Center for Wild Endangered Plant Resources, Shanghai Chenshan Botanical Garden, No.3888 Chenhua Road, Songjiang District, Shanghai 201602, China Shanghai Chenshan Botanical Garden Shanghai China; 8 Hangzhou Botanical Garden (Hangzhou West Lake Academy of Landscape Science), Hangzhou, Zhejiang 310000, China Hangzhou Botanical Garden Hangzhou China; 9 State Key Laboratory of Biocontrol and Guangdong Provincial Key Laboratory of Plant Resources, School of Life Sciences, Sun Yat-sen University, Guangzhou, Guangdong 510275, China Sun Yat-sen University Guangzhou China; 10 Key Laboratory of National Forestry and Grassland Administration on Plant Ex situ Conservation, Xiangshan-Wofosi Road, Beijing 100093, China Key Laboratory of National Forestry and Grassland Administration on Plant Ex situ Conservation Beijing China; 11 Beijing Botanical Garden, Beijing 100093, China Beijing Botanical Garden Beijing China

**Keywords:** Classification, lectotype, nomenclature, *Pourthiaea*, *Stranvaesia*, typification, *Weniomeles*

## Abstract

This study addresses the longstanding absence of a comprehensive phylogenetic backbone for the apple tribe Maleae, a deficiency attributed to limited taxon and marker sampling. We conducted an extensive taxon sampling, incorporating 563 plastomes from a diverse range of 370 species encompassing 26 presently recognized genera. Employing a range of phylogenetic inference methods, including RAxML and IQ-TREE2 for Maximum Likelihood (ML) analyses, we established a robust phylogenetic framework for the Maleae tribe. Our phylogenomic investigations provided compelling support for three major clades within Maleae. By integrating nuclear phylogenetic data with morphological and chromosomal evidence, we propose an updated infra-tribal taxonomic system, comprising subtribe Malinae Reveal, subtribe Lindleyinae Reveal, and subtribe Vauqueliniinae B.B.Liu (**subtr. nov.**). Plastid phylogenetic analysis also confirmed the monophyly of most genera, except for *Amelanchier*, *Malus*, *Sorbus* sensu lato, and *Stranvaesia*. In addition, we present a comprehensive taxonomic synopsis of *Photinia* and its morphological allies in the Old World, recognizing 27 species and ten varieties within *Photinia*, three species and two varieties within *Stranvaesia*, and two species and three varieties within *Weniomeles*. Furthermore, we also lectotypified 12 names and made two new combinations, *Photiniamicrophylla* (J.E.Vidal) B.B.Liu and *Weniomelesatropurpurea* (P.L.Chiu ex Z.H.Chen & X.F.Jin) B.B.Liu.

## ﻿Introduction

**Figure 1.**
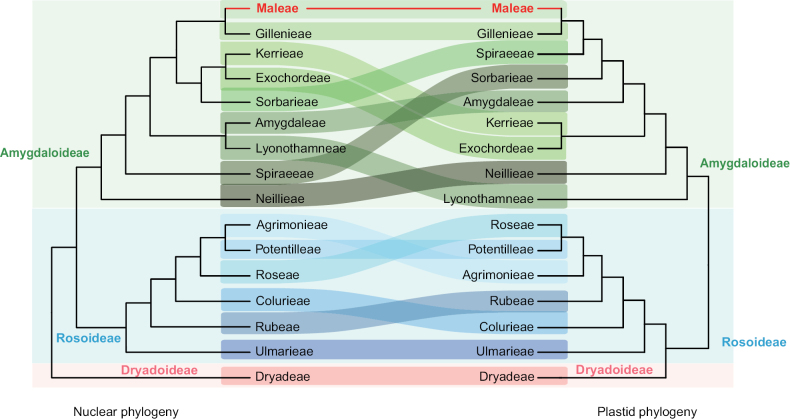
Infrafamilial- and tribe-level topological discordance within Rosaceae, highlighting the phylogenetic placement of the tribe Maleae **A** nuclear phylogeny based on transcriptome data (Xiang et al. 2017) **B** plastome-based phylogeny (Zhang et al. 2017).

The apple tribe Maleae, one of the sixteen tribes within the Rosaceae family, comprises approximately 27 genera and 912 species, with a widespread distribution across the Northern Hemisphere (Robertson et al. 1991; Lu et al. 2003; Phipps 2014). This tribe includes diverse genera such as *Kageneckia* Ruiz & Pav., *Lindleya* Kunth, and *Vauquelinia* Corrêa ex Bonpl., noted for their follicles and capsules, alongside pome-bearing genera previously categorized under the subfamily Maloideae (Morgan et al. 1994). The monophyly of this lineage has been confirmed by a series of phylogenetic studies (Fig. 1; Potter et al. 2007; Xiang et al. 2017; Zhang et al. 2017; Liu et al. 2020a, 2022).

As a prominent member of the nine tribes within the subfamily Amygdaloideae, the apple tribe Maleae has been consistently supported as a monophyletic group and the sister relationship to the tribe Gillenieae (Fig. 1). Within Maleae, numerous prior studies have consistently confirmed the close phylogenetic relationship between the dry-fruited genera (*Kageneckia*, *Lindleya*, and *Vauquelinia*) and the pome-bearing genera. This phylogenetic hypothesis has been corroborated by a series of studies employing a range of methods, from the utilization of singular or multiple plastid and nuclear markers (Morgan et al. 1994; Evans et al. 2000; Evans and Campbell 2002; Evans and Dickinson 2005; Verbylaitė et al. 2006; Campbell et al. 2007; Potter et al. 2007; Li et al. 2012; Lo and Donoghue 2012; Sun et al. 2018) to the most recent phylogenomic approaches (Xiang et al. 2017; Zhang et al. 2017; Liu et al. 2019, 2020a, 2022; Jin et al. 2023; Zhang et al. 2023). However, despite these endeavors, earlier phylogenetic studies were unable to resolve the intergeneric relationships within Maleae due to the limited plastid and nuclear markers. For instance, early studies by Campbell et al. (2007) and Potter et al. (2007) grouped the dry-fruited and pome-bearing genera under the tribe Pyreae (also known as Maleae). They also reclassified the pome-bearing genera (formerly known as subfamily Maloideae) into the subtribe Pyrinae (or Malinae). Despite these developments, the precise phylogenetic relationships and taxonomic status of *Kageneckia*, *Lindleya*, and *Vauquelinia* remained unresolved. Recent advancements in phylogenomics have demonstrated that datasets encompassing plastomes and/or hundreds of nuclear genes can offer sufficient informative sites for elucidating phylogenetic relationships. However, the substantial costs for genome-level sequencing have led to limited taxon sampling in contemporary phylogenomic analyses, such as the studies by Liu et al. (2022), Jin et al. (2023), and Zhang et al. (2023).

Accurately resolving its genus-level phylogenetic relationships has also remained a significant challenge. This difficulty is primarily attributed to the lack of informative genetic markers and ample taxon sampling, as highlighted in studies by Lo and Donoghue (2012) and Liu et al. (2022). During the Sanger sequencing era, Lo and Donoghue (2012) made a substantial contribution by assembling a dataset comprising 486 individuals, representing 331 species across 27 currently recognized genera. This dataset, one of the largest of its kind, utilized 11 plastid regions and one nuclear ribosomal internal transcribed spacer (nrITS) sequence. However, advancements in genetic research have revealed that such limited informative sites from several plastid and nuclear regions are insufficient for estimating a robust phylogenetic backbone. Next-generation sequencing (NGS) technologies, combined with decreasing sequencing costs and user-friendly bioinformatics tools, have revolutionized the approach to understanding phylogenetic relationships. The transition from Sanger sequencing to NGS has allowed for deeper phylogenetic analysis. A notable example of this progress is the study of Zhang et al. (2017), who estimated a plastid framework for the Rosaceae family using 122 plastomes, including 41 species from the Maleae tribe. This study marked a significant step in our evolutionary understanding of Maleae. Following this, there has been a surge of global research efforts to elucidate the phylogenetic relationships within Maleae using plastome-level datasets. Pioneering studies by Liu et al. (2019, 2020a, 2020b, 2022), Meng et al. (2021), Ulaszewski et al. (2021), Liu et al. (2023a, 2023b), Jin et al. (2023, 2024), and Ma et al. (2023) have significantly contributed to this field. These studies have employed extensive plastome datasets, vastly improving upon previous efforts in scale and depth. However, a common limitation of these studies has been the relatively narrow focus on a few species or a specific lineage within Maleae. This has resulted in an incomplete phylogenetic picture of Maleae. More comprehensive and inclusive research is needed, as it would provide a more thorough understanding of Maleae. Such an approach would involve extensive sampling across the tribe, incorporating a wide range of species to cover the full breadth of its genetic diversity.

The chloroplast genome, assembled from genome skimming data (Straub et al. 2012), has played a pivotal role in plant systematics and phylogenetics (Guo et al. 2023). Its highly conserved nature and areas of variable sequences make it widely used in phylogenetic analysis (Gitzendanner et al. 2018). Furthermore, this genetic stability, along with the non-recombinant of plastomes and often uniparental inheritance, offers a consistent and reliable framework for studying plant lineage and evolution, and the plastome-based phylogenetic inference has been successfully utilized in exploring the shallow (Zhang et al. 2017; Liu et al. 2019, 2020a, 2020b; Wang et al. 2020; Su et al. 2021) and deep phylogenies (Li et al. 2019, 2021). In this study, we assembled 563 plastomes from genome skimming data to reconstruct a comprehensive plastome-based phylogenetic framework for the tribe Maleae.

The taxonomic delimitation and phylogenetic relationship between *Photinia* Lindl. and its morphologically related genera in the Old World have been a subject of debate for centuries. In the Old World, the *Photinia*-affiliated genera comprised four groups: the deciduous genus *Pourthiaea* Decne., and the evergreen genera *Photinia*, *Stranvaesia* Lindl., and *Weniomeles* B.B.Liu. *Photinia* was initially described with a single evergreen species, *P.arbutifolia* Lindl., and later expanded to include four evergreen species (Lindley 1821). Subsequently, de Candolle (1825) incorporated two deciduous species into *Photinia*, thereby establishing the genus *Photinia*, encompassing both evergreen and deciduous species. *Photinia* has been recognized as comprising about 60 species, both evergreen and deciduous, distributed disjointedly across East and Southeast Asia, and Mexico (Rehder 1940; Vidal 1965; Yu 1974; Phipps et al. 1990; Robertson et al. 1991; Phipps 1992; Lu et al. 2003). Decaisne (1874) observed distinctive warty peduncles and pedicels on the fruits of deciduous species, setting them apart from their evergreen counterparts, leading to the establishment of these deciduous species under the newly formed genus *Pourthiaea*. This classification, recognizing *Pourthiaea* as a separate genus, gained widespread acceptance among botanists, including Nakai (1916), Ohashi (1989), Iketani and Ohashi (1991, 2001), Liu and Hong (2016a, 2016b, 2017), and Liu et al. (2023b). The separate generic status of *Pourthiaea* has also been further substantiated by recent molecular studies (Guo et al. 2011; Li et al. 2012; Zhang et al. 2017; Sun et al. 2018; Liu et al. 2019, 2022). Furthermore, Phipps (1992) revealed that the five species and three varieties of *Photinia* indigenous to Central America exhibit distinct morphological characteristics compared to the *Photinia* species from East Asia. This distinction was corroborated by phylogenomic evidence, which employed whole plastome and nuclear ribosomal DNA (nrDNA) datasets. Based on these findings, these Central American species were reclassified into a newly proposed genus, *Phippsiomeles* B.B.Liu & J.Wen, as elaborated in Liu et al. (2019).

**Figure 2.**
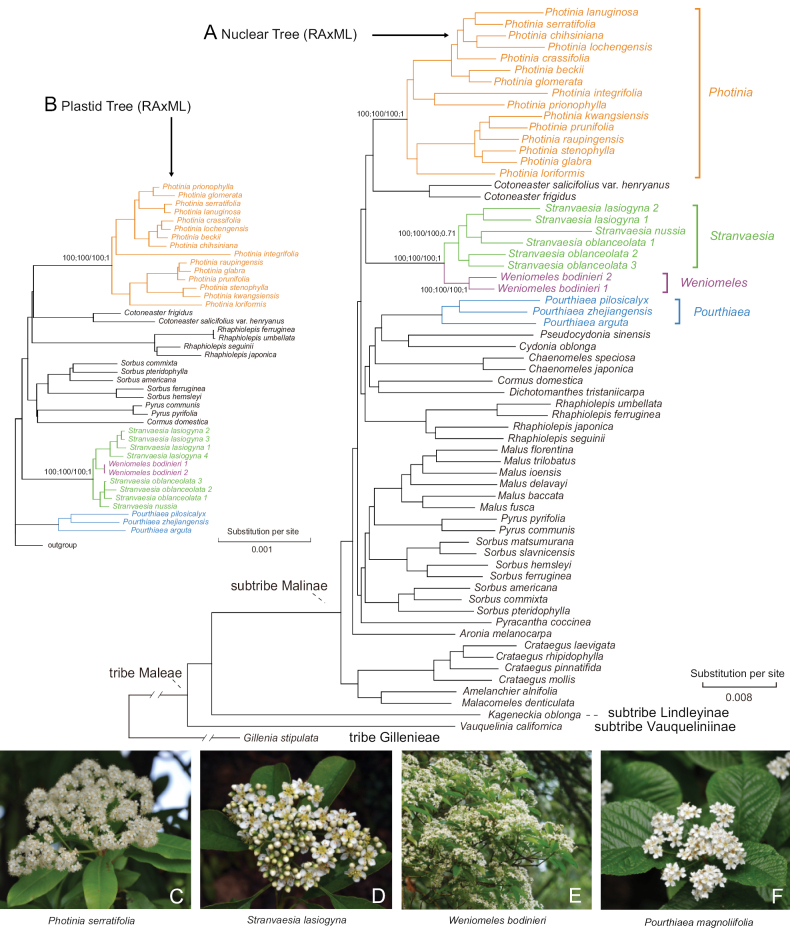
Phylogenetic tree of the apple tribe Maleae estimated by Maximum Likelihood (ML) algorithm using RAxML, based on a concatenated 426 single-copy nuclear genes (SCN genes) supermatrix **A** inset in the upper left corner **B** a segment of the RAxML tree focusing on *Photinia* and its allies, inferred from concatenated 78 plastid coding sequences (plastid CDSs). (Adapted from Jin et al. (2023)) **C***Photiniaserratifolia* (Zhejiang, China: Bin-Bin Liu) **D***Stranvaesialasiogyna* (Yunnan, China: Bin-Bin Liu) **E***Weniomelesbodinieri* (Yunnan, China: Bin-Bin Liu) **F***Pourthiaeamagnoliifolia* (Zhejiang, China: Bin-Bin Liu).

First described by Lindley in 1837, the red-fruit genus *Stranvaesia* is a relatively small group, encompassing five species native to China, the Himalayas, and Southeast Asia (Lu et al. 2003). Morphologically similar to *Photinia*, *Stranvaesia* is distinguishable by its unique characteristics, including a four- or five-chambered ovary and dehiscent fruits. These distinct features have led to its classification as a separate genus in numerous taxonomic studies spanning from the mid-19^th^ to early 21^st^ centuries (Roemer 1847; Decaisne 1874; Wenzig 1883; Focke 1888; Koehne 1893; Rehder 1940, 1949; Yu 1974; Lu et al. 2003). However, this classification was challenged by Kalkman (1973), who observed negligible differences in the number of carpels between *Stranvaesia* and *Photinia*. He noted that the supposedly dehiscent fruits of *Stranvaesiadavidiana* Decne. did not exhibit dehiscence in botanical garden observations, leading to the proposal of merging *Stranvaesia* into *Photinia* due to these morphological similarities. Despite this, the relationship between these two genera has been a long-standing taxonomic puzzle, with some botanists advocating for their distinct genus status (Yu 1974; Lu et al. 2003), while others supported merging them (Lu et al. 1991; Li et al. 1992; Zhang and Baas 1992). Recent phylogenetic and phylogenomic studies have shed light on this controversy. For instance, based on two chloroplast DNA regions and nrITS sequence, Guo et al. (2011) inferred that *Photiniadavidsoniae* Rehder & E.H.Wilson (= *P.bodinieri* H.Lév.) and *P.nussia* (Buch.-Ham. ex D.Don) Kalkman (= *Stranvaesianussia* (Buch.-Ham. ex D.Don) Decne.) formed a clade with strong support; however, the phylogenetic relationship between this clade and *Photinia* has been uncertain due to the limited informative sites. Liu et al. (2019) expanded the taxon sampling in their phylogenomic study within the Maleae framework, providing strong support for a redefined *Stranvaesia* clade, including three species, *S.bodinieri* (H.Lév.) B.B.Liu & J.Wen, *S.oblanceolata* (Rehder & E.H.Wilson) Stapf, and *S.nussia* (type species). Additionally, Liu et al. (2019) identified a novel distinguishing character for *Stranvaesia* not previously used in differentiating it from *Photinia*: the presence of a cluster of sclereids forming an ellipsoid between carpels in the flesh of pomes. This discovery, alongside the robust phylogeny, led to a redefinition of the generic limits of *Stranvaesia* and several nomenclatural changes. Further molecular analysis by Guo et al. (2020) confirmed the distinct phylogenetic placement of *Stranvaesia* and introduced another distinguishing trait: the unarmed branches of young trees. Despite this progress, ongoing uncertainties in the generic delimitation of *Photinia* and *Stranvaesia* persist due to factors like insufficient sampling (Liu et al. 2019) and limited informative sites (Guo et al. 2011, 2020). The complexity is compounded by polyploidy and hybridization-driven lineages, which challenge traditional taxonomic treatments. Jin et al. (2023) provided further insights, suggesting that the origin of the redefined genus *Stranvaesia* may involve allopolyploidy and introgression, with the most recent common ancestor (MRCA) of *Stranvaesiabodinieri* likely acting as the maternal parent and an extinct lineage as the paternal parent. Consequently, *Stranvaesiabodinieri* was proposed as a new genus, *Weniomeles*, characterized by purple-black fruits, thorny trunks and/or branches, and a fruit core with multiloculars separated by a sclereid layer and a sclereid cluster at the top of the locules (Fig. 2A).

Our study focuses on three key goals: 1) to establish a robustly plastome-based phylogenetic backbone for the apple tribe Maleae, 2) to update and refine the infra-tribal taxonomic system within Maleae, and 3) to compile a detailed taxonomic synopsis of *Photinia* and its closely related groups in the Old World.

## ﻿Materials and methods

### ﻿Taxon sampling, DNA extraction, and sequencing

In this study, we compiled 563 plastomes to estimate a plastid framework for the apple tribe Maleae. This collection encompasses 559 individuals within Maleae, representing almost all genera except for the Madeira endemic genus, *Chamaemeles* Lindl. We employed *Gillenia* Moench, comprising two species from four individuals, as the outgroup. Our dataset included 559 ingroup samples, covering a wide spectrum of species diversity across various genera: 14 out of 24 species in *Amelanchier* Medik. (14 individuals), all two species in *Aronia* Medik. (two individuals), all four species in *Chaenomeles* Lindl. (seven individuals), 63 taxa (53 species, eight varieties, one subspecies) out of 261 species in *Cotoneaster* Medik. (66 individuals), 20 out of 222 species in *Crataegus* L. (33 individuals), one species for the monotypic genus *Cydonia* Mill. (two individuals), one species for the monotypic genus *Dichotomanthes* Kurz (two individuals), six out of 11 species in *Hesperomeles* Lindl. (six individuals), one species for the monotypic genus *Heteromeles* M.Roem. (two individuals), all four species in *Kageneckia* Ruiz & Pav. (four individuals), one species for the monotypic genus *Lindleya* Kunth (one individual), one out of five species in *Malacomeles* (Decne.) Decne. (two individuals), three species in *Osteomeles* Lindl. (three individuals), one species for the monotypic genus *Peraphyllum* Nutt. (two individuals), three out of five species in *Phippsiomeles* (three individuals), 20 out of 27 species in *Photinia* (31 individuals), 14 species in *Pourthiaea* (53 individuals), one species for the monotypic genus *Pseudocydonia* (C.K.Schneid.) C.K.Schneid. (three individuals), three species in *Pyracantha* M.Roem. (five individuals), 17 out of 83 species in *Pyrus* L. (26 individuals), 30 out of 42 species in *Rhaphiolepis* Lindl. (40 individuals), all three species in *Stranvaesia* (three individuals), five species in *Vauquelinia* Corrêa ex Bonpl. (five individuals), and one species in *Weniomeles* (three individuals). Notably, we sampled 46 species and five cultivars in *Malus* Mill. (94 individuals) and 99 species out of 160 in *Sorbus* L. sensu lato (142 individuals), encompassing subgroups like *Aria* (Pers.) Host, *Chamaemespilus* Medik., *Cormus* Spach, *Micromeles* Decne., *Torminalis* Medik., and *Sorbus* sensu stricto. This comprehensive survey thus provides a significant insight into the plastid diversity of the Maleae tribe, covering a broad range of species and varieties across its numerous genera (Table 1).

Total genomic DNAs were extracted from silica-gel dried leaves and herbarium specimens using a modified cetyltrimethylammonium bromide (CTAB) method, as described by Li et al. (2013). This extraction was performed at the State Key Laboratory of Plant Diversity and Specialty Crops, Institute of Botany, Chinese Academy of Science (IBCAS) in China. The subsequent library preparation and sequencing processes were conducted at the Novogene laboratory in Beijing, utilizing the NEBNext^®^ Ultra™ II DNA Library Prep Kit, designed specifically for the Illumina^®^ platform. We generated paired-end reads of 150 bp using the Illumina HiSeq 2500 Instrument (Novogene Beijing). This approach ensured high-quality DNA sequencing, which is important for our research objectives.

### ﻿Plastome assembly and annotation

In our study, we adopted the Successive Approach combining Reference-based and De novo assembly (SARD approach: Liu et al. 2021, 2023b; Jin et al. 2024), a method offering the possibility of obtaining nearly all plastome-related reads, thus facilitating the production of high-quality chloroplast genomes even from datasets with low coverage. For initial data preparation, we used Trimmomatic v. 0.33 (Bolger et al. 2014) for quality trimming and adapter removal, complemented by FastQC v. 0.11.8 (Andrews 2018) for quality assessment. We then employed NOVOPlasty v. 4.3.3 (Dierckxsens et al. 2016), a *de novo* assembly program known for its accuracy and efficiency. The seed sequence chosen was the ribulose-1,5-bisphosphate carboxylase/oxygenase large subunit (*rbc*L), a 600 bp plastome-specific sequence with absence in the mitochondrial genome, to initiate the assembly process. While NOVOPlasty performs well for the deeply sequenced data, the SARD approach is notably effective even with lower-quality raw data. For the assembly process with SARD approach, all plastome-related reads were aligned to a reference genome using Bowtie2 (Langmead and Salzberg 2012), followed by generating a consensus sequence through Geneious Prime (Kearse et al. 2012). Concurrently, a *de novo* assembly was conducted using SPAdes v. 3.13.1 (Bankevich et al. 2012), which included error correction and employed a range of K-mer lengths (21, 33, 55, 77). The final step involved aligning scaffolds from the de novo assembly and contigs from NOVOPlasty to the draft plastome, and this step will effectively correct errors and ambiguities introduced from the first step, yielding a high-quality complete plastome.

We annotated the assembled plastid genomes using the PGA tool (Qu et al. 2019) with a closely related plastome as a reference. This process was followed by a thorough manual review of the coding sequences. We then translated these sequences into proteins using Geneious Prime to confirm the accuracy of the start and stop codons. To precisely delineate the boundaries of the large-single copy (LSC), small-single copy (SSC), and inverted repeats (IRs) regions, we employed the Find Repeats function in Geneious Prime based on the characteristic presence of two reverse complementary repeats in the plastomes of Rosaceae species. After this detailed annotation process, we converted our custom annotations into the format required for NCBI submissions. This involved creating both FASTA files and five-column feature tables, a task we accomplished using the GB2sequin tool (Lehwark and Greiner 2019).

### ﻿Data matrix generation and sequence cleaning

Our previous studies have consistently shown that phylogenetic trees derived from entire plastome datasets and the 79 concatenated plastid protein-coding sequences (plastid CDSs) yield almost identical topologies within the apple tribe framework (Liu et al. 2020a, 2020b, 2022). This similarity underscores the minimal influence of potential misalignments in the intron regions. Consequently, we opted to utilize the whole plastome for phylogenetic inference in this study. To mitigate systematic errors stemming from alignment inaccuracies, we applied trimAL v. 1.2 (Capella-Gutiérrez et al. 2009) to fine-tune the alignment of the plastome. Additionally, we incorporated Spruceup (Borowiec 2019) to identify, visualize, and eliminate outlier sequences. In this process, we set a window size of 50 and an overlap of 25, ensuring a rigorous and precise approach to enhance the quality and reliability of our phylogenetic analysis.

### ﻿Phylogenomic analyses based on various inference methods

In our comprehensive study, we implemented a variety of robust inference methodologies to achieve precise and reliable phylogenetic results. Initially, we employed PartitionFinder2 (Stamatakis 2006; Lanfear et al. 2016) to identify the most appropriate partitioning schemes and molecular evolution models, utilizing its default settings. This critical step ensured that the chosen models and schemes were best suited for our dataset, enhancing the accuracy of our subsequent analyses.

For estimating Maximum Likelihood (ML) trees, we utilized the advanced capabilities of IQ-TREE2 v. 2.2.0.3 (Minh et al. 2020), conducting analyses with 1000 SH-aLRT and ultrafast bootstrap replicates. This method provided us with a robust statistical framework to evaluate the reliability of the phylogenetic tree branches. In parallel, we used RAxML v. 8.2.12 (Stamatakis 2014), adopting the GTRGAMMA model for each partition. This process included running 200 rapid bootstrap replicates to support the clade structures in our phylogenetic tree, thus ensuring a comprehensive and reliable assessment of clade support.

### ﻿Nomenclatural synopsis and typification

Over 11 years, from 2013 to 2023, we conducted an in-depth taxonomic study to examine all names published under the genus *Photinia* and its related genera. This comprehensive review was not a trivial undertaking; it involved a thorough exploration of multiple renowned online botanical databases. These included Tropicos (accessible at https://www.tropicos.org), the International Plant Names Index (IPNI) at https://www.ipni.org/, and The Plant List, available at http://www.theplantlist.org/. Our investigation extended beyond these databases to encompass a wide range of literature pertinent to the genus *Photinia*, ensuring no relevant information was overlooked.

## ﻿Results

### ﻿A plastid phylogenetic backbone of *Photinia* and allies

We newly generated 147 complete plastomes for this study, and we collected 563 plastomes representing 370 species to create a detailed phylogenetic framework for the apple tribe. Our efforts resulted in a comprehensive aligned plastome matrix that was used for ML analyses. This matrix, spanning a significant length of 158,752 base pairs, was curated with poorly aligned regions being carefully trimmed to ensure the accuracy of our phylogenetic inferences.

**Figure 3.**
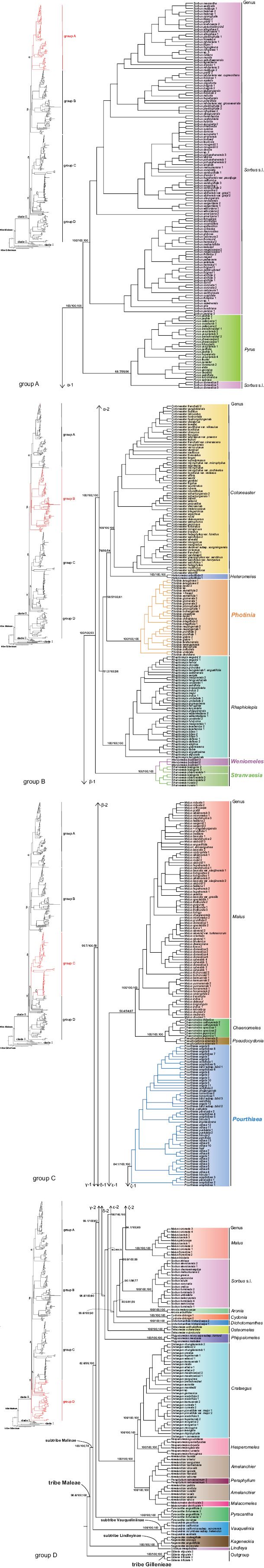
A comprehensive phylogenetic backbone of the apple tribe Maleae, including 563 plastomes across 370 species and 26 genera, estimated by IQ-TREE2 based on the whole plastome dataset. Each of the 26 genera is represented by a unique color for clear distinction. Owing to the extensive scope of the tree, it is segmented into four distinct groups (labeled Group A, B, C, and D), each depicted in separate images. The interconnections among these subgroups are denoted by branch connectors labeled α, β, γ, δ, ε, and ζ.

We successfully generated two phylogenetic trees using the ML method, i.e., RAxML and IQ-TREE trees. All these phylogenetic trees consistently corroborated the monophyly of three major clades within the apple tribe (Fig. 3, Suppl. materials 1, 2). Clade I, identified as the most basal of the three, comprises two genera: *Lindleya* and *Kageneckia*. This clade lays the foundation of our phylogenetic understanding of the tribe. Clades II and III, on the other hand, demonstrate a sister relationship to each other and, collectively, they are sister to Clade I. Clade II is uniquely composed of a single genus, *Vauquelinia*, highlighting its distinct evolutionary path within the tribe. Clade III is particularly noteworthy as it corresponds to what was previously known as the subfamily Maloideae, encompassing approximately 24 genera. This finding solidifies the genetic distinctiveness of these genera within the apple tribe. However, there were notable exceptions, including *Amelanchier*, *Malus*, *Sorbus* s.l., and *Stranvaesia*.

## ﻿Discussion

### ﻿Refining the phylogenetic backbone with plastome data: towards an updated infra-tribal classification of Maleae

In our study, we integrated representative species from three dry-fruited genera—*Kageneckia*, *Lindleya*, and *Vauquelinia*—alongside a comprehensive sampling of pome-bearing genera to estimate their maternally phylogenetic relationships. The inferred plastid phylogeny (Fig. 3, Suppl. materials 1, 2) corroborated the monophyly of these groups, each representing distinct subtribes within Maleae. Furthermore, this topology indicates a clear successive sister relationship between a combined clade (*Kageneckia* + *Lindleya*) and *Vauquelinia*, relative to the pome-bearing genera. Morphologically, these three clades can be easily distinguished, a distinction further elaborated in the identification key provided later.

**Figure 4.**
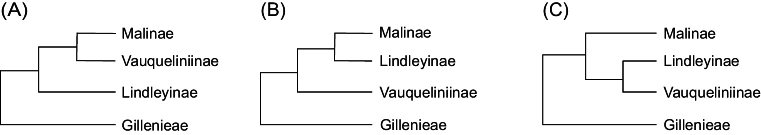
Phylogenetic hypotheses among subtribes within the apple tribe Maleae. **A** plastome-based topology (current study; Liu et al. 2020a); 11 plastid regions- and nuclear ribosomal internal transcribed spacer (nrITS)-based topology (*atp*B-*rbc*L, *psb*A-*trn*H, *rbc*L, *rpl*16 intron, *rpl*20-*rps*12, *rps*16 intron, *trn*C-*ycf*6, *trn*G-*trn*S, *trn*H-*rpl*2, *trn*L-*trn*F, and *trn*K + *mat*K; Lo and Donoghue 2012); transcriptome-based topology (Xiang et al. 2017; Zhang et al. 2023) **B** single-copy nuclear genes (SCN genes)-based topology (inferred from Maximum Likelihood (ML) methods: Liu et al. 2022; Jin et al. 2023) **C**SCN genes-based topology (ASTRAL-III species tree: Liu et al. 2022; Jin et al. 2023).

However, the phylogenetic relationships among these subtribes have been subject to variability across different studies leveraging diverse genomic datasets (Fig. 4). Phylogenies inferred from transcriptomic data (Xiang et al. 2017; Zhang et al. 2023) reveal a topology similar to the plastome-based topology analyses among these three subtribes, i.e., combined clades of Vauqueliniinae and Malinae together sister to Lindleyinae (Fig. 4A). Conversely, recent phylogenomic studies employing ML inference method with hundreds of single-copy nuclear genes (SCN genes) datasets–785 genes in Liu et al. (2022) and 426 genes in Jin et al. (2023)—have elucidated an alternative phylogenetic hypothesis, (Malinae, Lindleyinae) Vauqueliniinae (Fig. 4B). In contrast, a species tree inferred through a coalescent-based method (Jin et al. 2023) presents a unique topology, i.e., the sister relationship between Lindleyinae and Vauqueliniinae, and then together sister to Malinae (Fig. 4C). Despite the emergence of three divergent topologies, the monophyly of these three clades has been consistently supported across multiple previous studies. This convergence underscores the robustness of this newly proposed infra-tribal taxonomic classification within tribe Maleae, despite the methodological diversity and inherent complexities of phylogenomic analysis.

While the maternally inherited characteristics of plastomes in the Maleae tribe obviate the need for orthology inference, their utility is somewhat limited in identifying hybridization and polyploidization events (McKain et al. 2018; Guo et al. 2023). The complex evolutionary processes within Maleae, such as hybridization, polyploidization, and incomplete lineage sorting, have profoundly influenced its origin and diversification. This is evident from a series of phylogenomic studies that highlight cytonuclear discordance within the tribe (refer to Fig. 2A, B and studies by Liu et al. 2022; Hodel et al. 2023; Jin et al. 2023; Zhang et al. 2023). However, the phylogenetic topologies inferred from hundreds of SCN genes, as illustrated in our previous studies (Liu et al. 2022; Jin et al. 2023), lend strong support to the three major clades identified in our plastid tree (Fig. 3, Suppl. materials 1, 2). These findings have led us to formally propose a taxonomic system for the tribe Maleae, delineating it into three subtribes, i.e., subtribe Lindleyinae, subtribe Malinae, and subtribe Vauqueliniinae. Consequently, this study not only elucidates the phylogenetic placement of these dry-fruited genera within the tribe but also significantly contributes to refining their taxonomy.

#### ﻿Tribe Maleae Small, Man. S.E. Fl. 632. 1933. Type: Malus

Taxon classificationPlantaeRosalesRosaceae

Mill.

F32EEA9A-4D31-53C8-88D3-9157EB5A3E22

= Pyreae Baill., Hist. Pl. 1: 442, 475. 1869. Type: Pyrus L.

### ﻿Key to subtribes of Maleae

**Table:** 

**1a**	Leaf margins not horny; carpels ± adnate to hypanthium; flowers: perianth and androecium epigynous; fruit pome; seed not winged or pyrenes; Northern Hemisphere, rarely extending to Central America; 2n = 34	**subtribe Malinae**
**1b**	Leaf margins usually horny; carpels free; flowers: perianth and androecium perigynous; Fruit woody capsule or follicle; seed winged; Central & South America; 2n = 30 or 34	**2**
**2a**	Fruit capsule or follicle; seed 2 or many; 2n = 34	**subtribe Lindleyinae**
**2b**	Fruit capsule; seed 2; 2n = 30	**subtribe Vauqueliniinae**

#### ﻿1. Subtribe Malinae

Taxon classificationPlantaeRosalesRosaceae

Reveal, Phytoneuron 2012-33: 2. 2012.

F1E626F9-2643-5F41-B324-596EEFE32B82

≡ Malaceae Small, Fl. S.E. U.S. [Small]. 529. 1903, nom. cons. Type: Malus Mill.

##### Remark.

This tribe contains ca. 24 genera (ca. 905 species), *Amelanchier* (24 species), *Aronia* (two species), *Chaenomeles* (four species), *Chamaemeles* (one species), *Cotoneaster* (261 species), *Crataegus* (222 species), *Cydonia* (one species), *Dichotomanthes* (one species), *Hesperomeles* (11 species), *Heteromeles* (one species), *Malacomeles* (five species), *Malus* (33 species), *Osteomeles* (two species), *Peraphyllum* (one species), *Phippsiomeles* (five species), *Photinia* (27 species), *Pourthiaea* (seven species), *Pseudocydonia* (one species), *Pyracantha* (six species), *Pyrus* (83 species), *Rhaphiolepis* (42 species), *Sorbus* s.l. (*Chamaemespilus*, *Aria*, *Torminalis*, *Cormus*, *Micromeles*, and *Sorbus* s.s.; ca. 160 species), *Stranvaesia* (three species), and *Weniomeles* (two species). 2n = 34.

#### ﻿2. Subtribe Lindleyinae

Taxon classificationPlantaeRosalesRosaceae

Reveal, Phytoneuron 2012-37: 217. 2012.

FEB6FEE5-1C81-5CA5-837B-082837E87B7D

≡ Lindleyaceae J.Agardh, Theoria Syst. Pl. 166. 1858. Type: Lindleya Kunth., nom. cons.

##### Remark.

This subtribe contains two genera, *Lindleya* (one species) and *Kageneckia* (ca. three species), distributed in Central and South America. 2n = 34.

#### ﻿3. Subtribe Vauqueliniinae

Taxon classificationPlantaeRosalesRosaceae

B.B.Liu subtr. nov.

D944DB99-74A9-5B1E-81EB-497725D37890

urn:lsid:ipni.org:names:77342732-1

##### Type.

***Vauquelinia*** Corrêa ex Bonpl.

##### Description.

Large shrubs or small trees, evergreen. Leaves simple, coriaceous, with serrate margins. Inflorescences terminal, 15–25+-flowered, compound corymbs. Flowers bisexual, 5-merous. Hypanthium hemispherical. Sepals 5, erect, broadly ovate, valvate. Petals 5, white, oblong-ovate to oblong-obovate. Stamens 18-20. Carpels 5, free from hypanthium, ventrally connate; ovules 2 per cell, ascending, apotropous. Fruits capsules, broadly ovoid, sericeous, ventrally (fully) and dorsally (in distal 1/2) dehiscent, splitting into 5 follicles; hypanthium persistent; sepals persistent, erect; styles persistent. Seeds 2 per follicle. 2n = 30.

##### Remark.

This subtribe comprises only one genus, *Vauquelinia*, with about three species distributed in Mexico and the Southwestern United States.

### ﻿A taxonomic synopsis of *Photinia* and its morphological allies in the Old World

Within the Old World, the genus *Photinia* and its morphologically allied genera can be classified into four distinct clades. These include the deciduous genus *Pourthiaea* and three evergreen genera: *Photinia*, *Stranvaesia*, and *Weniomeles*, as redefined in recent studies (Liu et al. 2019; Jin et al. 2023). This study undertook the most extensive taxonomic sampling to date and inferred a well-supported phylogenetic backbone of these four genera in the framework of the tribe Maleae based on the whole plastome. This finding suggests that the evergreen genus *Photinia* is closely related to a clade combining *Heteromeles* and *Cotoneaster*, the deciduous genus *Pourthiaea* is sister to the transatlantic group of *Malus*, and *Weniomeles* is phylogenetically nested within *Stranvaesia*. Contrarily, the recent transcriptome-based nuclear phylogeny (Zhang et al. 2023) suggested an alternative phylogenetic relationship, positioning *Photinia* alongside *Heteromeles*, and *Pourthiaea* sister to a group of genera characterized by multiple ovules, including *Chaenomeles*, *Cydonia*, and *Pseudocydonia*. It is noteworthy that Zhang et al. (2023) did not include any species of *Stranvaesia* and *Weniomeles* in their sampling. Addressing this sampling gap, the phylogenomic investigation by Jin et al. (2023) elucidated the close phylogenetic relationship between *Stranvaesia* and *Weniomeles*, which, in turn, collectively form a sister clade to a group comprising *Photinia* and *Cotoneaster*. The significant cytonuclear discordance revealed the potential reticulation events in the origin of these genera.

Nomenclaturally, the genus *Pourthiaea* has been thoroughly evaluated, including 213 names in a comprehensive checklist (Lou et al. 2022). In this study, we focus on the remaining three evergreen genera: *Photinia*, *Stranvaesia*, and *Weniomeles*. We aim to conduct an in-depth nomenclature assessment and typification for these genera. This entails a critical review of the existing names, verification of their validity according to botanical nomenclature rules, and clarification of type specimens for each taxon. Our analysis aims to provide clarity and precision in the taxonomic classification of these genera, contributing to a better understanding of their evolutionary relationships and aiding in their accurate identification and study in botanical and ecological research.

#### ﻿ Photinia

Taxon classificationPlantaeRosalesRosaceae

Lindl., Bot. Reg. 6: t. 491. 1820. nom. cons.

814CE76C-B0D2-5D96-A1F7-48573EE55C45

##### Type.

*Photiniaserrulata* Lindl., nom. illeg. ≡ *Crataegusglabra* Thunb. ≡ *Photiniaglabra* (Thunb.) Franch. & Sav., type conserved by Nesom and Gandhi (2009).

##### Remark.

Approximately 27 species and 10 varieties are found across East, South, and Southeast Asia.

#### ﻿1. Photinia anlungensis

Taxon classificationPlantaeRosalesRosaceae

T.T.Yu, Acta Phytotax. Sin. 8: 228. 1963.

7CE06229-DCA1-5386-93FC-23547CD6C278

≡ Pyrus anlungensis (T.T.Yu) M.F.Fay & Christenh., Global Fl. 4: 95. 2018.

##### Type.

China. Guizhou: Anlong, 15 June 1960, *C.S. Chang & Y.T. Chang 5359* (holotype: PE [barcode 00061327!]; isotype: HGAS [barcode 021155!]).

##### Distribution.

China (Guizhou).

#### ﻿2. Photinia beckii

Taxon classificationPlantaeRosalesRosaceae

C.K.Schneid., Ill. Handb. Laubholzk. [C.K.Schneider] 1: 707. 1906.

2A20D758-31F2-5EBB-B336-379B3B9A330F

≡ Pyrus beckii (C.K.Schneid.) M.F.Fay & Christenh., Global Fl. 4: 98. 2018.

##### Type.

China. Yunnan: Mengtze, woods, 5500 feet, *A. Henry 9795A* (lectotype, designated by Pathak et al. (2021: 39): E [barcode E00010996!]; isolectotypes: A [barcode 00045594!], US [barcode 00097493!]). Image of lectotype available from https://plants.jstor.org/stable/10.5555/al.ap.specimen.e00010996.

##### Distribution.

China (Yunnan).

#### ﻿3. Photinia berberidifolia

Taxon classificationPlantaeRosalesRosaceae

Rehder & E.H.Wilson, Pl. Wilson. (Sargent) 1(2): 191. 1912.

6592A387-2415-53D8-872D-658E1CAE92BA

≡ Pyrus berberidifolia (Rehder & E.H.Wilson) M.F.Fay & Christenh., Global Fl. 4: 98. 2018.

##### Type.

China. Sichuan, Tung Valley, May 1904, *E.H. Wilson 3508* (holotype: A [barcode 00038561!]; isotypes: A [barcode 000385610!], K [barcode K000758250!]). Image of holotype available from https://plants.jstor.org/stable/10.5555/al.ap.specimen.a00038561.

##### Distribution.

China (Sichuan).

#### ﻿4. Photinia chihsiniana

Taxon classificationPlantaeRosalesRosaceae

K.C.Kuan, Acta Phytotax. Sin. 8(3): 227. 1963.

867AF8E8-01BE-5975-86EA-6416ED7D33ED

≡ Pyrus chihsiniana (K.C.Kuan) M.F.Fay & Christenh., Global Fl. 4: 100. 2018.

##### Type.

China. Guangxi: Lingui, 8 May 1950, *C.S. Chung 808097* (holotype: IBK [barcode IBK00062054!]; isotypes: GAC [barcode GAC0010558], IBSC [barcode 0004364!], PE [barcode 00299791!]). ibidem, 22 November 1953, *C.F. Liang 31096* (paratypes: GAC [barcode GAC0010567!], IBSC [barcode 0004332!], KUN [barcode 607115!], PE [barcode 00299793!], SYS [barcode sys00075317!]). Lingui, Yanshan, 20 April 1951, *C.S. Chung 808829* (paratypes: GAC [barcode GAC0010559!], IBSC [barcode 0318308!], PE [barcode 00299794!]). ibidem, *C.S. Chung 808871* (paratypes: GAC [barcode GAC0010557!], IBK [barcode IBK00062057!, IBK00062205!], IBSC [barcode 0318305!, 0318306!]). ibidem, 23 July 1950, *C.S. Chung 808679* (paratypes: GAC [barcode GAC0010573!], IBK [barcode IBK00062224!], IBSC [barcode 0318307!]). Pinglou, 23 April 1958, *Z.Z. Chen 52327* (paratypes: IBK [barcode IBK00062052!, IBK00190808!], IBSC [barcode 0335042!], KUN [barcode 607345!]). Guilin, 8 July 1937, *W.T. Tsang 27773* (paratypes: IBSC [barcode 0318304!], SYS [barcode SYS00074928!]). ibidem, August 1937, *W.T. Tsang 27992* (paratypes: IBSC [barcode 0318303!], SYS [barcode sys00095740!]). ibidem, 29 March 1948, *C.N. Tang 13423* (paratype: IBK [barcode IBK00062056!]).

##### Distribution.

China (Guangxi and Hunan).

#### ﻿5. Photinia chingiana

Taxon classificationPlantaeRosalesRosaceae

Hand.-Mazz., Sinensia 2: 125. 1932.

A7C0E1AF-B8C1-57E1-91A9-DE540916C9AB

≡ Pyrus chingiana (Hand.-Mazz.) M.F.Fay & Christenh., Global Fl. 4: 100. 2018.

##### Type.

China. Kwangsi (Guangxi, Yishan): Bui-tung, Nibai ad conf. prov. Kweichou, 1000 m, in silvis apertis vel ripis rivorum, raro, 27 June 1928, *R.C. Ching 6244* (lectotype, designated by Pathak et al. (2021: 39): NY [barcode NY00436112!; isolectotypes: IBSC [barcode 0004365!], NAS [barcode NAS00071252!, NAS00071253!], PE [barcode 00026318!]). Image of lectotype available from https://plants.jstor.org/stable/10.5555/al.ap.specimen.ny00436112.

##### Distribution.

China (Guangxi and Guizhou).

#### ﻿5a. Photinia chingiana var. chingiana

Taxon classificationPlantaeRosalesRosaceae

B2C0909C-4CF3-5929-A1BF-DBE344758485

= Photinia austroguizhouensis Y.K.Li, Bull. Bot. Res., Harbin 6(4): 107. 1986. Type: CHINA. Guizhou: Libo, *M.Z. Yang et al. 810333* (holotype: HGAS; isotype: PE [barcode 01432751!]).
= Photinia simplex Y.K.Li & X.M.Wang, Bull. Bot. Res., Harbin 8(3): 133. 1988. Type: CHINA. Guizhou: Sandu County, Yaorenshan, *Y.K. Li 10173* (holotype: HGAS; isotype: PE [barcode 01432750!]).

##### Distribution.

China (Guangxi and Guizhou).

#### ﻿5b. Photinia chingiana var. lipingensis

Taxon classificationPlantaeRosalesRosaceae

(Y.K.Li & M.Z.Yang) L.T.Lu & C.L.Li, Acta Phytotax. Sin. 38(3): 277. 2000.

D96D6A8F-5895-5064-9E39-6E0D24E49468

≡ Photinialipingensis Y.K.Li & M.Z.Yang, Bull. Bot. Res., Harbin 8(3): 134. 1988.

##### Type.

China. Guizhou: Liping, Zhongchao, October 1987, *D.F. Huang 714* (holotype: HGAS; isotype: PE [barcode 01432752!]).

##### Distribution.

China (Guizhou).

#### ﻿6. Photinia chiuana

Taxon classificationPlantaeRosalesRosaceae

Z.H.Chen, Feng Chen & X.F.Jin, J. Hangzhou Univ., Nat. Sci. Ed. 20(1): 32. 2021.

7429E0BA-56E8-5020-90FA-4F944D87FB6D

##### Type.

China. Zhejiang: Qujiang, Hunan Town, Poshi Village, Bijiashanzhuang, alt. 140 m, 20 May 2019, *Z.H. Chen, L. Chen, & Q.S. Lin QJ19052001* (holotype: ZM; isotype: ZM).

##### Distribution.

China (Zhejiang).

#### ﻿7. Photinia crassifolia

Taxon classificationPlantaeRosalesRosaceae

H.Lév., Flore du Kouy-Tchéou 349. 1915.

77965B5A-638A-5010-A2E2-4B9471853C61

≡ Pyrus crassifolia (H.Lév.) M.F.Fay & Christenh., Global Fl. 4: 101. 2018.
= Photinia cavaleriei H.Lév., Repert. Spec. Nov. Regni Veg. 11: 66. 1912. later homonym. non H.Lév., Repert. Spec. Nov. Regni Veg. 4: 334. 1907. Type: CHINA. Guizhou: Tin-fan (= Huishui), June 1909, *J. Cavalerie 3571* (holotype: E [barcode E00011309!]). Image of holotype available from https://plants.jstor.org/stable/10.5555/al.ap.specimen.e00011309.
= Photinia crassifolia var. denticulata Cardot, Notul. Syst. (Paris) 3: 372. 1918. Type: CHINA. Guizhou, San-chouen (= Anshun), 1910, *J. Cavalerie 3571-pp* (lectotype, designated by Pathak et al. (2021: 39): P [barcode P02143157!]; isotype: P [barcode P02143156!]). Image of lectotype available from https://plants.jstor.org/stable/10.5555/al.ap.specimen.p02143157.

##### Type.

China. Guizhou: Gan-chouen (= Anshun), April 1912, *J. Cavalerie 3571* (lectotype, designated by Pathak et al. (2021: 39): E [barcode E00284677!]; isolectotype: P [barcode P02143158!]). Image of lectotype available from https://plants.jstor.org/stable/10.5555/al.ap.specimen.p02143158.

##### Distribution.

China (Guangxi, Guizhou, and Yunnan).

#### ﻿8. Photinia cucphuongensis

Taxon classificationPlantaeRosalesRosaceae

T.H.Nguyên & Yakovlev, Bot. Zhurn. (Moscow & Leningrad) 65(9): 1351 (in error as 1251). 1980.

D42F4B9C-EE96-584E-A054-1796B4F6AF7D

≡ Pyrus cucphuongensis (T.H.Nguyên & Yakovlev) M.F.Fay & Christenh., Global Fl. 4: 101. 2018.

##### Type.

Vietnam. Ninh Binh: Cuc Phuong, 29 January 1975, *A.L. Takhtadjan & N.T. Hiep 8565* (holotype: LE; isotype: HN).

##### Distribution.

Vietnam.

#### ﻿9. Photinia davidiana

Taxon classificationPlantaeRosalesRosaceae

(Decne.) Cardot, Bull. Mus. Natl. Hist. Nat. 25(5): 399. 1919.

91E9CA50-0C98-50FB-9CCC-8412830955B8

≡ Stranvaesia davidiana Decne., Nouv. Arch. Mus. Hist. Nat. 10: 179. 1874.

##### Type.

China. Tibet: Baoxing, Mou-Pin “now belongs to Sichuan”, 1870, *A. David s.n.* (holotype: P [barcode P02143103!]). Image of holotype available from https://plants.jstor.org/stable/10.5555/al.ap.specimen.p02143103.

#### ﻿9a. Photinia davidiana var. davidiana

Taxon classificationPlantaeRosalesRosaceae

55A0AFB1-71C1-5CFF-8FCA-A9076077AADA

**Figure 5.**
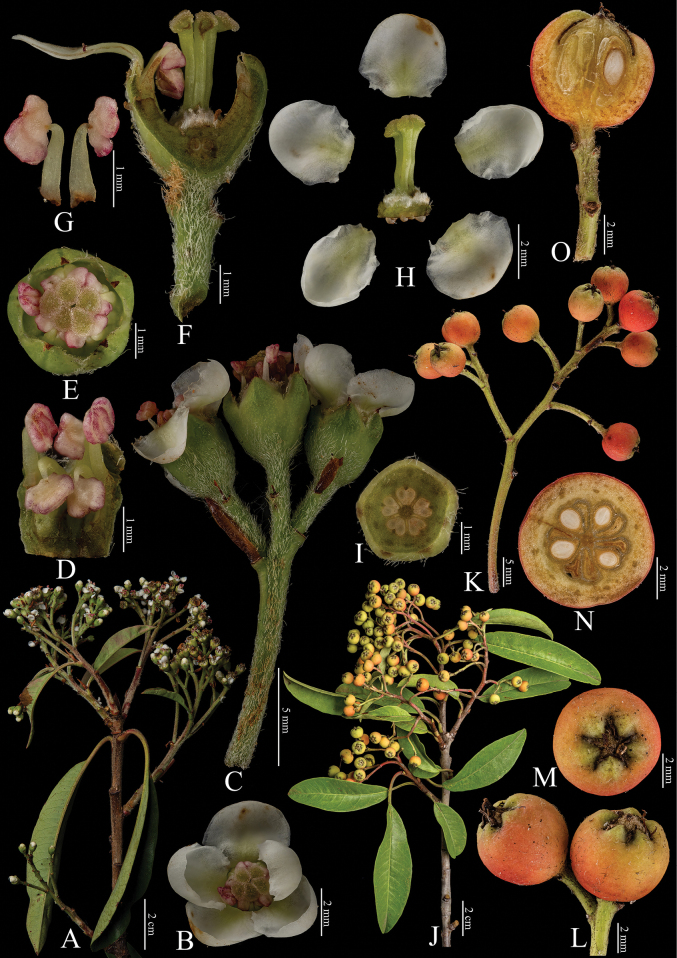
Fine structure of *Photiniadavidiana*, encompassing various developmental stages and perspectives. **A** inflorescence branch **B** top view of a single flower **C** inflorescence branchlet **D, G** stamens **E** top view of an unopened flower **F** longitudinal section through the ovary **H** dissected flower showing internal structures **I** cross-section of the immature ovary **J** infructescence branch **K** infructescence branchlet **L** mature fruit **M** fruit, viewed from above **N** cross-sections of fruit **O** longitudinal section of fruit. The inflorescence branches (**A–I**) were collected on April 15, 2024, while the infructescence branches (**J–O**) were gathered on October 7, 2023. Yan-Li Wen was responsible for the collection of all fresh specimens at the Kunming Institute of Botany, Chinese Academy of Sciences (Yunnan, China). Furthermore, Bin-Jie Ge (Chenshan Botanical Garden, Shanghai, China) dissected and photographed all the samples.

Fig. 5 Common name: 红豆果树(原变种)(Chinese name)

= Stranvaesia integrifolia Stapf, Hooker’s Icon. Pl. 23: t. 2295. 1894. ≡ Photiniahavilandii Stapf, Bot. Mag. 149: sub t. 9008. 1924, replacement name. Type: MALESIA. Borneo: Kinabalu, *G.D. Haviland 1071* (holotype: K [barcode K000758362!]; isotypes: K [barcode K000758363!], BM [barcode BM000602185!]). Image of holotype available from https://plants.jstor.org/stable/10.5555/al.ap.specimen.k000758362.
= Stranvaesia henryi Diels, Bot. Jahrb. Syst. 36(5, Beibl. 82): 52. 1905. Type: CHINA. Sichuan, February 1890, *A. Henry 8953* (lectotype, designated by Vidal (1965: 232): K [barcode K000758304!]). Image of lectotype available from https://plants.jstor.org/stable/10.5555/al.ap.specimen.k000758304.
= Photinia niitakayamensis Hayata, J. Coll. Sci. Imp. Univ. Tokyo 30(1): 103. 1911. ≡ Stranvaesianiitakayamensis (Hayata) Hayata, Icon. Pl. Formosan. 8: 33. 1919. Type: CHINA. Taiwan: Chiayi, Yushan, Mt. Niitaka, *S. Nagasawa 551* (**lectotype, designated here**: KYO [barcode KYO00022357!]; isolectotype: KYO [barcode KYO00022358!]).
= Pyrus cavaleriei H.Lév., Repert. Spec. Nov. Regni Veg. 11: 67. 1912. Type: CHINA. Guizhou: Pin-Fa, *J. Cavalerie 3569* (holotype: P [barcode P02143101!]; isotypes: A [barcode 00045576!], E [barcode E00011338!, E00284670!], P [barcode P02143100!, P02143102!]). Image of holotype available from https://plants.jstor.org/stable/10.5555/al.ap.specimen.p02143101.
= Photinia undulata var. formosana Cardot, Notul. Syst. (Paris) 3: 372. 1914. ≡ Photiniadavidianavar.formosana (Cardot) H.Ohashi & Iketani, J. Jap. Bot. 69(1): 22. 1994. Type: CHINA. Formose (Taiwan): Arisan (Alishan), *L.U. Faurie 77* (lectotype, designated by Wang et al. (2018: 90): P [barcode P02143109!]). Image of lectotype available from https://plants.jstor.org/stable/10.5555/al.ap.specimen.p02143109.
= Photinia davidiana f. latifolia Cardot, Bull. Mus. Natl. Hist. Nat. 25(5): 399. 1919. Type: CHINA. Yunnan: bois de Kou-toui, au-dessus de Mo-so-yn, *J.M. Delavay 3978* (holotype: L [barcode 1901178!]).
= Stranvaesia salicifolia Hutch., Bot. Mag. 146: t. 8862. 1920. ≡ Stranvaesiadavidianavar.salicifolia (Hutch.) Rehder, J. Arnold Arbor.7(1): 29. 1926. Type: CHINA. Hupeh (Hubei): north and south of Ichang, alt. 1300–2000 m, October 1907, *E.H. Wilson 382a* (**lectotype, designated here**: A [barcode 00045607!]). Image of lectotype available from https://plants.jstor.org/stable/10.5555/al.ap.specimen.a00045607.

##### Distribution.

China (Gansu, Guangxi, Guizhou, Hubei, Jiangxi, Shaanxi, Sichuan, Taiwan, Yunnan) and Malaysia (Kinabalu).

#### ﻿9b. Photinia davidiana var. undulata

Taxon classificationPlantaeRosalesRosaceae

(Decne.) LongY.Wang, W.Guo & W.B.Liao, Phytotaxa 361(1): 91. 2018.

14552F25-9776-558A-AA3B-E40042A61C78

≡ Stranvaesia undulata Decne., Nouv. Arch. Mus. Hist. Nat. 10: 179. 1874. ≡ Eriobotryaundulata (Decne.) Franch., Pl. Delavay. 226. 1890. ≡ Photiniaundulata Cardot, Bull. Mus. Natl. Hist. Nat. 25: 399. 1919. ≡ Stranvaesiadavidianavar.undulata (Decne.) Rehder & E.H.Wilson, Pl. Wilson. 1(2): 192. 1912.
= Stranvaesia davidiana var. suoxiyuensis C.J.Qi & C.L.Peng, J. Wuhan Bot. Res. 7(3): 239. 1989. Type: CHINA. Hunan: Cili, *C.L. Peng & C.L. Long 120358* (holotype: CSFC).

##### Type.

China. Kouy-Tcheou (= Guizhou): *Perny s.n.* (holotype: P [barcode P02143104!]; isotype: P [barcode P02143105!]). Image of holotype available from https://plants.jstor.org/stable/10.5555/al.ap.specimen.p02143104.

##### Distribution.

China (Fujian, Guangxi, Guizhou, Hubei, Hunan, Jiangxi, Shaanxi, Sichuan, Yunnan, and Zhejiang) and Vietnam (Tonkin).

#### ﻿10. Photinia glabra

Taxon classificationPlantaeRosalesRosaceae

(Thunb.) Franch. & Sav., Enum. Pl. Jap. 1(1): 141. 1873.

7DB0644A-FB92-50BB-B6CA-55CDE35ECD02

≡ Crataegus glabra Thunb., Syst. Veg., ed. 14 (J. A. Murray). 465. 1784. ≡ Mespilusglabra Poir., Encycl. [J. Lamarck & al.] 4(2): 446. 1798. ≡ Photiniaserrulata Lindl., Trans. Linn. Soc. London 13: 103, t. 10 (1821), nom. illeg. ≡ Photiniaglabra (Thunb.) Poit., Rev. Hort. (Paris) 11: 228. 1849. ≡ Photiniaglabra (Thunb.) Maxim., Bull. Acad. Imp. Sci. Saint-Pétersbourg 19(2): 178. 1873, isonym. ≡ Photiniaglabra (Thunb.) Decne., Nouv. Arch. Mus. Hist. Nat. 10: 140. 1874, isonym. ≡ Pyrusthunbergii M.F.Fay & Christenh., Global Fl. 4: 123. 2018.
= Photinia glabra var. typica Maxim., Bull. Acad. Imp. Sci. Saint-Pétersbourg 19(2): 179. 1873.

##### Type.

Japan. Kanname, *Thunberg 11860* (syntype). ibidem, *Thunberg 11861* (syntype).

##### Distribution.

China (Anhui, Fujian, Guangdong, Guangxi, Guizhou, Hubei, Hunan, Jiangsu, Jiangxi, Sichuan, Yunnan, and Zhejiang), Japan, Myanmar, Thailand, and Vietnam.

#### ﻿11. Photinia griffithii

Taxon classificationPlantaeRosalesRosaceae

Decne., Nouv. Arch. Mus. Hist. Nat. 10: 142. 1874.

97B4515F-55D3-5B85-AC57-FE6E91C753C7

**Figure 6.**
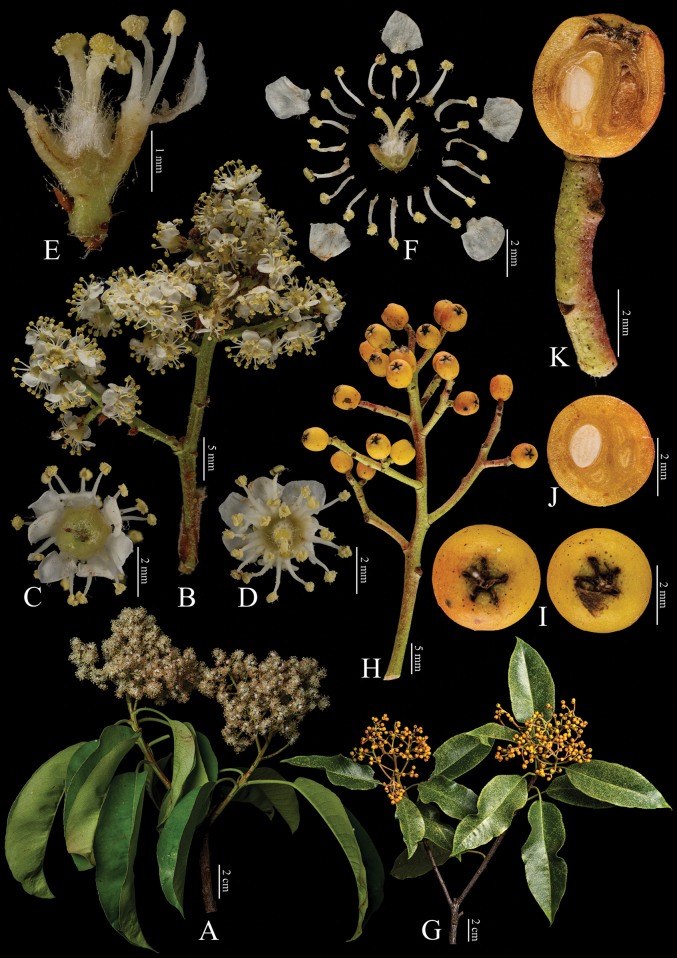
Fine structure of *Photiniagriffithii*, encompassing various developmental stages and perspectives. **A** inflorescence branch **B** inflorescence branchlet **C** bottom perspective of an individual flower **D** top view of a single flower **E** longitudinal section through the ovary **F** dissected flower showing internal structures **G** an infructescence branch **H** infructescence branchlet **I** fruit, viewed from above **J** cross-sections of fruit **K** longitudinal section of fruit. The inflorescence branches (**A–F**) were collected on April 15, 2024, while the infructescence branches (**G–K**) were gathered on October 7, 2023. Yan-Li Wen was responsible for the collection of all fresh specimens at the Kunming Institute of Botany, Chinese Academy of Sciences (Yunnan, China). Furthermore, Bin-Jie Ge (Chenshan Botanical Garden, Shanghai, China) dissected and photographed all the samples.

Fig. 6 Common name: 球花石楠 (Chinese name); pinyin (spelled as sounds in Chinese): qiu hua shi nan

≡ Eriobotrya griffithii (Decne.) Franch., Pl. Delavay. 1: 224. 1890. ≡ Photiniaserrulatavar.congestiflora Cardot, Notul. Syst. (Paris) 3: 373. 1918. nom. superfl. ≡ Pyrusgriffithiana M.F.Fay & Christenh.; Global Fl. 4: 105. 2018.
= Photinia glomerata Rehder & E.H.Wilson, Pl. Wilson. (Sargent) 1(2): 190. 1912. ≡ Pyrusglomerata (Rehder & E.H.Wilson) M.F.Fay & Christenh., Global Fl. 4: 105. 2018. Type: CHINA. Yunnan, Szemao, *A. Henry 11716* (lectotype, selected by Vidal (1965: 226), first step; second step, designated by Wang et al. (2019: 599): E [barcode E00011310!]; isolectotypes: A [barcode 00038560!], K [barcode K000758251!], MO [barcode MO-255089!], US [barcode 00097496!]). *A. Henry 11716A* (syntypes: US [barcode 00097497!], A [barcode 00045567!, 00045568!], E [barcode E00284676!], K [barcode K000758252!], MO [barcode MO-255088!]). Image of lectotype available from https://plants.jstor.org/stable/10.5555/al.ap.specimen.e00011310.
= Photinia franchetiana Diels, Notes Roy. Bot. Gard. Edinburgh 5: 272. 1912. Type: CHINA. Yunnan, *G. Forrest 487* (holotype: E [barcode E00011311!]). Image of holotype available from https://plants.jstor.org/stable/10.5555/al.ap.specimen.e00011311
= Photinia glomerata Rehder & E.H.Wilson var. cuneata T.T.Yu, Acta Phytotax. Sin. 8(3): 227. 1963. Type: CHINA. Yunnan, Yung-jen, *H.T. Tsai 52879* (holotype: PE [barcode 00336359!]; isotypes: IBSC [barcode 0318765!], PE [barcode 00336360!], A [barcode 00137699!], NAS [barcode NAS00071255!], KUN [barcode 608247!]).
= Photinia glomerata Rehder & E.H.Wilson var. microphylla T.T.Yu, Acta Phytotax. Sin. 8(3): 227. 1963. Type: CHINA. Yunnan, Teng-chuan, Mt. Chih-shan, *R.C. Ching 24894* (holotype: PE [barcode 00336361!]; isotypes: PE [barcode 00336291!], KUN [barcode 607608!]).
= Photinia semiserrata H.Li, Fl. Dulongjian Reg. 131. 1993, nom. nud.

##### Type.

Bhutan. Himalaya orientalis, 1837–1838, *Griffith 2087* (lectotype, designated by Wang et al. (2019: 599): P [barcode P02143170!]; isotypes: K [barcode K000758185!], L [barcode L0019505!], M [barcode M-0213887!]). Image of lectotype available from https://plants.jstor.org/stable/10.5555/al.ap.specimen.p02143170.

##### Distribution.

Bhdan and China (Hubei, Sichuan, and Yunnan).

#### ﻿12. Photinia integrifolia

Taxon classificationPlantaeRosalesRosaceae

Lindl., Trans. Linn. Soc. London 13(1): 103, t. 10. 1821.

43491439-452F-5818-A1EE-28B2D92C548E

≡ Eriobotrya integrifolia (Lindl.) Kurz, J. Asiat. Soc. Bengal, Pt. 2, Nat. Hist. 45(4): 304. 1877. ≡ Pyrusintegrifolia (Lindl.) M.F.Fay & Christenh., Global Fl. 4: 108. 2018.

##### Type.

Nepal. 7 November 1821, *Wallich 669* (lectotype, selected by Kalkman (1973: 419) ‘holotype’, first step; second step, designated by Pathak et al. (2019: 184): K [barcode K001111555!]; isolectotypes: E [barcode E00011312!], GH [barcode 00045579!], GZU [barcode 000283019!], K [barcode K000758314!, K001111556!], L [barcode L0019506!, L0019507!], P [barcode P02143206!], NY [barcode 00436120!]). Image of lectotype available from https://plants.jstor.org/stable/10.5555/al.ap.specimen.k001111555.

##### Distribution.

Bangladesh, Bhutan, China (Guangxi, Guizhou, Tibet, Yunnan), India (Arunachal Pradesh, Assam, Manipur, Meghalaya, Sikkim, Tamil Nadu, Uttar Pradesh, West Bengal), Indonesia (Gunung Ulu Kali, Pahan, Java, Lesser Sunda Isl.), Laos, Myanmar (Chin, Kachin, Mandalay, Sagaing), Nepal, Thailand, and Vietnam.

#### ﻿12a. Photinia integrifolia var. integrifolia

Taxon classificationPlantaeRosalesRosaceae

4F406442-4EC8-5A5E-862D-0BA0EB5A787F

**Figure 7.**
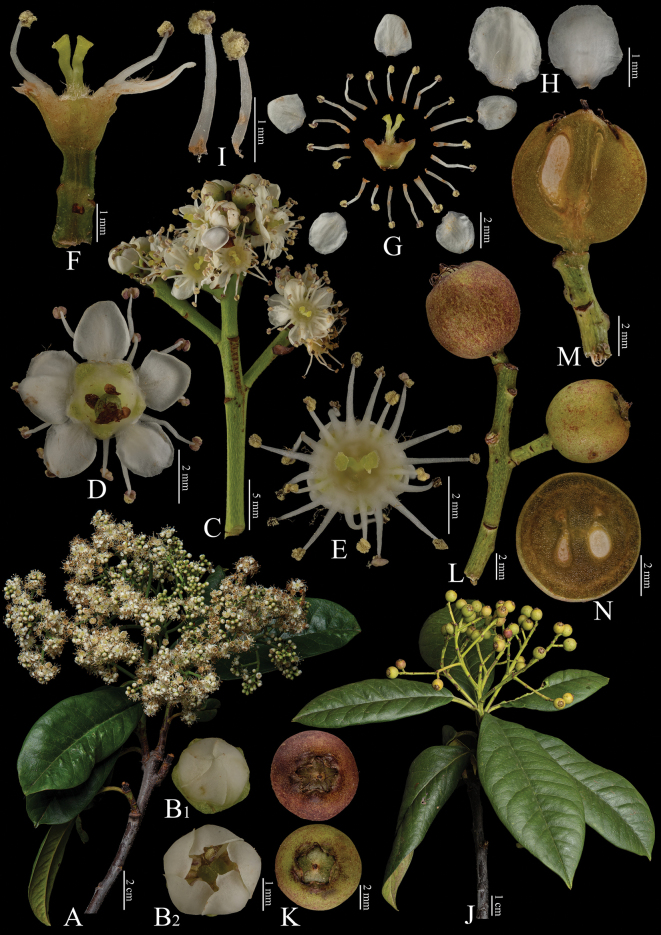
Fine structure of *Photiniaintegrifolia*, encompassing various developmental stages and perspectives. **A** inflorescence branch **B1** top view of an unopened flower **B2** top view of an opening flower **C** inflorescence branchlet **D** bottom perspective of an individual flower **E** top view of a single flower with the absence of petals **F** longitudinal section through the ovary **G** dissected flower showing internal structures **H** petals **I** stamens **J** an infructescence branch **K** fruit, viewed from above **L** infructescence branchlet **M** longitudinal section of fruit **N** cross-sections of fruit. The inflorescence branches (**A–I**) were collected on April 15, 2024, while the infructescence branches (**J–N**) were gathered on October 7, 2023. Yan-Li Wen was responsible for the collection of all fresh specimens at the Kunming Institute of Botany, Chinese Academy of Sciences (Yunnan, China). Furthermore, Bin-Jie Ge (Chenshan Botanical Garden, Shanghai, China) dissected and photographed all the samples.

Fig. 7 Common name: 全缘石楠(原变种)(Chinese name)

= Pyrus integerrima Wall. ex D.Don, Prodr. Fl. Nepal. 237. 1825, nom. illeg. superfl. ≡ Photiniaintegerrima (Wall. ex D.Don) N.P.Balakr., Fl. Jowai 1: 191. 1981.
= Photinia scandens Stapf, Bot. Mag. 149: sub t. 9008. 1924. ≡ Stranvaesiascandens (Stapf) Hand.-Mazz., Symb. Sin. 7(3): 483. 1933. Type: CHINA. Yunnan: Shweli-Salwin divide, *G. Forrest 9329* (holotype: E [barcode E00011339!]; isotypes: K [barcode K000758309!], IBSC [barcode 0318894!]). Image of holotype available from https://plants.jstor.org/stable/10.5555/al.ap.specimen.e00011339.
= Photinia myriantha Merr., Brittonia 4: 82. 1941. Type: MYANMAR. Adung Valley, *F.K.Ward 9276* (holotype: A [barcode 00026802!]); Ngawchang Valley, near Black Rock, *F.K. Ward 359* (paratype: NY [barcode 00436121!]). Image of holotype available from https://plants.jstor.org/stable/10.5555/al.ap.specimen.a00026802.
= Photinia integrifolia var. yunnanensis T.T.Yu, Acta Phytotax. Sin. 8(3): 229. 1963. Type: CHINA. Yunnan: Wei-si, alt. 2500 m, *K.M. Feng 4167* (holotype: PE [barcode 00004602!]; isotypes: PE [barcode 00336524!, 00336554!], KUN [barcode 607497!]). Kung-shan (Champutung) alt. 1600–1800 m, *K.M. Feng 8153* (paratypes: PE [barcode 00336477!, 00336552!]).

##### Distribution.

Bangladesh, Bhutan, China (Guangxi, Guizhou, Tibet, Yunnan), India, Indonesia, Myanmar, Nepal, Thailand, and Vietnam.

#### ﻿12b. Photinia integrifolia var. flavidiflora

Taxon classificationPlantaeRosalesRosaceae

(W.W.Sm.) J.E.Vidal, Adansonia, n.s. 5: 227. 1965.

9205FA5F-AE21-526F-9716-092256A708BA

≡ Photinia flavidiflora W.W.Sm., Notes Roy. Bot. Gard. Edinburgh 10: 59. 1917.

##### Type.

China. Yunnan: Mingkwong Vally, November 1912, *G. Forrest 9221* (lectotype, designated by Vidal (1965: 227): E [barcode E00011313!]; isolectotype: A [barcode 00026742!]). Hills to the N. W. Tengyueh, *G. Forrest 9294* (syntypes: BM [barcode BM000602131!], E [barcode E00072939!], K [barcode K000758267!], A [barcode 00026743!]). Divide between the Tengyueh and Shweli Valleys, *G. Forrest 7901* (syntype). Image of lectotype available from https://plants.jstor.org/stable/10.5555/al.ap.specimen.e00011313.

##### Distribution.

China (Yunnan) and Myanmar (Kachin).

#### ﻿12c. Photinia integrifolia var. notoniana

Taxon classificationPlantaeRosalesRosaceae

(Wight & Arn.) J.E.Vidal, Addisonia 5: 227. 1965.

D31C7071-2CAA-5A7A-8F28-60C1DDB513E9

≡ Photinia notoniana Wall. ex Wight & Arn., Prodr. Fl. Ind. Orient. 1: 302. 1834. ≡ Eriobotryanotoniana (Wall. ex Wight & Arn.) Kurz, Prelim. Rep. Forest Pegu App. B. 48. 1875.
= Photinia eugenifolia Lindl., Edwards’s Bot. Reg. 23: t. 1956. 1837. ≡ Photinianotonianavar.eugenifolia Hooker, Fl. Brit. India 2: 381. 1878. Type: INDIA. Pundua, 1832, *Wallich 670B* (lectotype, designated by Vidal (1965: 226): K [barcode K001111558!]). Image of lectotype available from https://plants.jstor.org/stable/10.5555/al.ap.specimen.k001111558.
= Photinia micrantha Decne., Nouv. Arch. Mus. Hist. Nat. 10: 143. 1874. ≡ Photinianotonianaf.micrantha (Decne.) Koord. & Valeton, Bijdr. Boomsoort. Java 5: 364. 1900. Type: INDIA / BABGLADESH. Bengalia orientalis, *Griffith 2098* (lectotype, selected by Vidal (1965: 227), first step; second step, designated by Kalkman (1973: 420): K [barcode K000758325!]; isolectotype: P [barcode P02143138!]). Image of lectotype available from https://plants.jstor.org/stable/10.5555/al.ap.specimen.k000758325.
= Photinia notoniana var. ceylanica Hook.f., Fl. Brit. India 2: 381. 1878. Type: INDIA. *G. Walker s.n.* (lectotype, designated by Pathak et al. (2019: 185): K [barcode K000758326!]). Image of lectotype available from https://plants.jstor.org/stable/10.5555/al.ap.specimen.k000758326.
= Photinia notoniana var. macrophylla Hook.f., Fl. Brit. India 2: 381. 1878. Type: INDIA. Khasia Hills, *J.D. Hooker & T. Thomoson s.n.* (lectotype, designated by Pathak et al. (2019: 185): K [barcode K000758321!]; isolectotypes: K [barcode K000758319!, K000758322!, K000758323!]). Image of lectotype available from https://plants.jstor.org/stable/10.5555/al.ap.specimen.k000758321.
= Photinia sambuciflora W.W.Sm., Notes Roy. Bot. Gard. Edinburgh 10: 60. 1917. Type: CHINA. Yunnan: Hills to the north of Tengyueh, *G. Forrest 9722* (**lectotype**, selected by Vidal (1965: 227), first step; **second step, designated here**: E [barcode E00011314!]; isolectotypes: HBG [barcode HBG-511070!], BM [barcode BM000602132!]); Shweli-Salween divide, *G. Forrest 12293* (syntypes: BM [barcode BM000602133!], E [barcode E00072952!], K [barcode K000758268!]). Image of lectotype available from https://plants.jstor.org/stable/10.5555/al.ap.specimen.e00011314.

##### Type.

India. Nilghiris, *Wight 1014* (lectotype, selected by Vidal (1965: 226) ‘holotype’: K [barcode K000758317!]; isolectotypes: E [barcode E00011315!], P [barcode P02143139!]). Image of lectotype available from https://plants.jstor.org/stable/10.5555/al.ap.specimen.k000758317.

##### Distribution.

China (Yunnan), India, and Laos.

#### ﻿12d. Photinia integrifolia var. sublanceolata

Taxon classificationPlantaeRosalesRosaceae

Miq., Fl. Ned. Ind.1(1): 387. 1855.

BE52F8C4-D0DF-5BD6-AF10-CE94C4DC3F47

= Photinia integrifolia var. subdenticulata Miq., Fl. Ned. Ind.1(1): 387. 1855. Type: INDONESIA. Java: Mount Prahu, *T. Horsfield 1135* (lectotype, designated by Kalkman (1973: 420) ‘holotype’: K [barcode K000758360!]; isolectotype: BM [barcode BM000602182!]). Image of lectotype available from https://plants.jstor.org/stable/10.5555/al.ap.specimen.k000758360.
= Photinia dasythrysa Miq., Fl. Ned. Ind. 1(1): 387. 1855. ≡ Photiniaintegrifoliavar.dasythrysa (Miq.) J.E.Vidal, Adansonia 5: 227. 1965. Type: INDONESIA. Sumatra: Sunda-eilanden, *Miquel s.n.* (holotype: U [barcode U0123984!]). Image of holotype available from https://plants.jstor.org/stable/10.5555/al.ap.specimen.u0123984.
= Photinia notoniana var. angustata Blume ex K.Koch, Ann. Mus. Bot. Lugduno-Batavi 1: 250. 1864, nom. nud.
= Photinia blumei Decne., Nouv. Arch. Mus. Hist. Nat. 11: 142. 1874. Type: INDONESIA. Java, mons Malabar, 19 October 1861, *Anderson 83* (lectotype, designated by Vidal (1965: 227): P [barcode P02143205!]; isolectotype: K [barcode K000758361!]); *Wight 923* (syntype: P [barcode P02143136!]); *Wight 924* (syntype: P [barcode P02143137!]). Image of lectotype available from https://plants.jstor.org/stable/10.5555/al.ap.specimen.p02143205.
= Photinia notoniana f. grandiflora Koord. & Valeton, Bijdr. Boomsoort. Java 5: 364. 1900. Type: not designated.
= Photinia notoniana f. vulgaris Koord. & Valeton, Bijdr. Boomsoort. Java 5: 364. 1900. Type: not designated.

##### Type.

Indonesia. Java: Surakarta, *T. Horsfield 432* (lectotype, designated by Kalkman (1973: 420) ‘holotype’: K [barcode K000758357!]; isolectotype: BM [barcode BM000602183!]). Image of lectotype available from https://plants.jstor.org/stable/10.5555/al.ap.specimen.k000758357.

##### Distribution.

Indonesia (Java and Sumatra).

#### ﻿13. Photinia lanuginosa

Taxon classificationPlantaeRosalesRosaceae

T.T.Yu, Acta Phytotax. Sin. 8(3): 227. 1963.

DBADC848-7351-500C-9FC2-F95B8BD824AC

**Figure 8.**
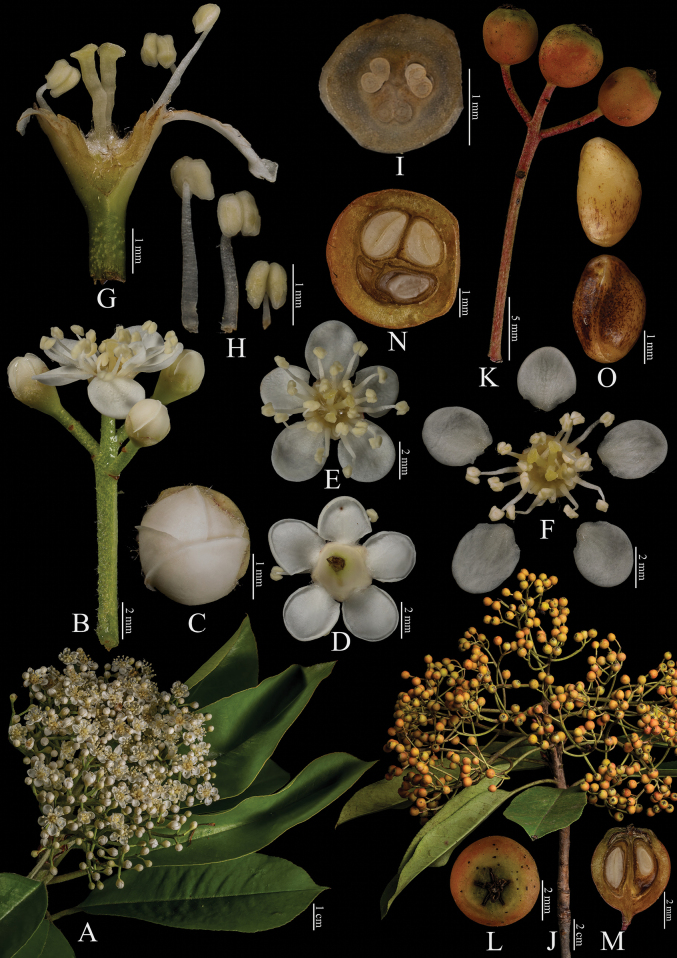
Fine structure of *Photinialanuginosa*, encompassing various developmental stages and perspectives. The inflorescence branches, depicted in **A–I**, include **A** whole branch **B** branchlet **C** top view of an unopened flower **D** bottom perspective of an individual flower **E** top view of a single flower **F** dissected flower showing internal structures **G** longitudinal section through the ovary **H** stamens **I** cross-section of the immature ovary. These were collected on April 3, 2024. The infructescence branches, shown in **J–O**, comprise: **J** whole branch **K** branchlet **L** top view of a developing fruit **M** longitudinal section of the fruit **N** cross-sections of the fruit **O** seed. These were collected on November 29, 2023. All fresh specimens were collected by Ting Wang at the Hangzhou Botanical Garden, Zhejiang, China. Additionally, Bin-Jie Ge from the Chenshan Botanical Garden in Shanghai, China, dissected and photographed all samples.

Fig. 8 Common name: 绵毛石楠 (Chinese name); pinyin (spelled as sounds in Chinese): mian mao shi nan

≡ Pyrus atalantae M.F.Fay & Christenh., Global Fl. 4: 96. 2018.

##### Type.

China. Hunan, Mt. Xuefengshan, *C.T. Li 1882* (holotype: PE [barcode 00026329!]; isotype: IBSC [barcode 0344338!], PE [barcode 00004601!]).

##### Distribution.

China (Hunan).

#### ﻿14. Photinia lindleyana

Taxon classificationPlantaeRosalesRosaceae

Wight & Arn., Prodr. Fl. Ind. Orient. 1: 302. 1834.

8C37431A-27E3-5934-A192-BC02EE633916

≡ Photinia serrulata var. lindleyana (Wight & Arn.) Wenz., Linnaea 38: 94. 1873. ≡ Pyruslindleyana (Wight & Arn.) M.F.Fay & Christenh., Global Fl. 4: 110. 2018.
= Photinia lindleyana var. tomentosa Gamble, Fl. Madras 1(3): 445. 1919. ≡ Photiniaserratifoliavar.tomentosa (Gamble) Vivek. & B.V.Shetty, Bull. Bot. Surv. India 23(3–4): 256. 1983. ≡ Pyruslindleyanavar.tomentosa (Gamble) K.S.Kumar & Arum., Indian Forester 148(1): 115. 2022. Type: INDIA. Tamil Nadu, Nilgiris District, between Bangi Tappal and Sispara, alt. 7500 ft. ASL, May 1889, *J.S. Gamble 20638* (lectotype, designated by Kumar and Arumugam (2022: 115): MH [barcode MH00234090!]).

##### Type.

India. Peninsula Ind. orientalis, *Wight 1012* (lectotype, selected by Kalkman (1973: 424), first step; second step, designated by Kumar and Arumugam (2022: 117): K [barcode K000758313!]; isolectotypes: BM [barcode BM000602140!], E [barcode E00011327!]). *Wight 1013* (syntypes: BM [barcode BM000602139!], E [barcode E00174590!, E00174591!], GZU [barcode GZU000283017!], K [barcode K000758312!], P [barcode P02143117!]). Image of lectotype available from https://plants.jstor.org/stable/10.5555/al.ap.specimen.k000758313.

##### Distribution.

China (Sichuan and Yunnan) and India (Kerala and Tamil Nadu).

#### ﻿14a. Photinia lindleyana var. lindleyana

Taxon classificationPlantaeRosalesRosaceae

E052299C-FFC3-579A-BF34-2CB7E9A115A4

##### Distribution.

China (Sichuan and Yunnan) and India (Kerala and Tamil Nadu).

#### ﻿14b. Photinia lindleyana var. yunnanensis

Taxon classificationPlantaeRosalesRosaceae

Cardot, Notul. Syst. (Paris) 3: 374. 1918.

6E1AD485-6AC5-5F66-98BC-956153B934FA

##### Type.

China. Yunnan: ao Kouy Chan près My Li, 1906, *F. Ducloux & P. Ngeou 4242-pp* (**lectotype, designated here**: P [barcode 02143143!]; isolectotype: P [barcode 02143144!]). Yunnan: Lan argy tsin, près Lou lan, 17 April 1908, *F. Ducloux & J.B. Lo 5936* (syntype: P [barcode P02143144!]).

##### Distribution.

China (Yunnan).

#### ﻿15. Photinia lochengensis

Taxon classificationPlantaeRosalesRosaceae

T.T.Yu, Acta Phytotax. Sin. 8(3): 226. 1963.

E0036E6B-8F2E-5B9A-9E02-7D5B2B95C5A1

≡ Pyrus lochengensis (T.T.Yu) M.F.Fay & Christenh., Global Fl. 4: 110. 2018.

##### Type.

China. Guangxi: Lo-cheng (=Luocheng), *W. Chen 84410* (holotype: IBSC; isotypes: PE [barcode 00004611!, 01790013!]). Note A.

##### Distribution.

China (Guangxi).

##### Note A.

In the protologue, Yu and Kuan (1963) designated the type specimen as being deposited in the herbarium “HC”, which they referenced as “Herb. Inst. Austro-Sin. Acad. Sin. Canton”. The correct standard name for this institute is the South China Botanical Garden (IBSC). However, we could not locate any specimens from this collection in IBSC. Instead, we found two isotype sheets at the PE herbarium.

#### ﻿16. Photinia loriformis

Taxon classificationPlantaeRosalesRosaceae

W.W.Sm., Notes Roy. Bot. Gard. Edinburgh 10: 60. 1917.

1F7CC29A-1476-58EC-9285-2131297F2B22

≡ Pyrus loriformis (W.W.Sm.) M.F.Fay & Christenh., Global Fl. 4: 111. 2018.

##### Type.

China. Yunnan, Yunnanfu (=Kunming), *E.E. Maire 1118* (**lectotype, designated here**: E [barcode E00011317!]; isolectotypes: A [barcode A00045580!], K [barcode K000758253!]). *E.E. Maire 1117* (syntype: E [barcode E00285982!]), *E.E. Maire 1755* (syntype: E [barcode E00285984!]), *E.E. Maire 2099* (syntype: E [barcode E00285985!]). Note B. Image of lectotype available from https://plants.jstor.org/stable/10.5555/al.ap.specimen.e00011317.

##### Distribution.

China (Sichuan and Yunnan).

##### Note B.

In the protologue, the author referenced four collections collected by *E.E. Maire: 1118, 1117, 1755, and 2099*, all housed in the herbarium E. However, Smith did not designate a specific type, meaning all four collections are syntypes. A lectotypification is required (Turland et al. 2018). Upon examination of each specimen from the herbarium E, it was observed that *E.E. Maire 1117* (barcode E00285982) and *1755* (barcode E00285984) lack flowers and fruits. *E.E. Maire 2099* (barcode E00285985) has fruits, but they are damaged by worms. As a result, *E.E. Maire 1118* (barcode E00011317), which is in good condition and has flowers, has been selected as the lectotype.

#### ﻿17. Photinia maximowiczii

Taxon classificationPlantaeRosalesRosaceae

Decne., Nouv. Arch. Mus. Hist. Nat. 10: 143. 1874.

F082488E-534C-5AA4-8C7E-36B73A9ABFE3

= Photinia wrightiana Maxim., Bull. Acad. Imp. Sci. Saint-Pétersbourg 32: 486. 1888. Type: JAPAN. Bonin-sima, *Wright s.n.* (syntype). Liukiu, *A. Tashiro s.n.* (syntype).

##### Type.

Japan. Bonin Islands, *Wright 80* (holotype: P [barcode P02143127!]; isotype: K [barcode K000758301!]). Image of holotype available from https://plants.jstor.org/stable/10.5555/al.ap.specimen.p02143127.

##### Distribution.

Japan (Bonin Islands and Liukiu).

#### ﻿18. Photinia megaphylla

Taxon classificationPlantaeRosalesRosaceae

T.T.Yu & L.T.Lu, Acta Phytotax. Sin. 18(4): 493. 1980.

9B473480-3DC0-5D6B-B823-279792CEA057

= Pyrus megaphylla (T.T.Yu & L.T.Lu) M.F.Fay & Christenh., Global Fl. 4: 111. 2018.

##### Type.

China. Tibet: Motuo, *Qingzang Exped. 74-4158* (holotype: PE [barcode 00026327!]).

##### Distribution.

China (Tibet).

#### ﻿19. Photinia microphylla

Taxon classificationPlantaeRosalesRosaceae

(J.E.Vidal) B.B.Liu comb. nov.

E0364C18-8012-5DCC-83A2-252E43EC21C9

urn:lsid:ipni.org:names:77342733-1

= Stranvaesia microphylla J.E.Vidal, Notul. Syst. (Paris) 13: 300. 1949. ≡ Pyruspluto M.F.Fay & Christenh., Global Fl. 4: 116. 2018.

##### Type.

Vietnam. Tonkin: massif du Lo Sui Tong, Près Chapa (Cha-pa and Cho-bo), 2200 m, 29 July 1926, *E. Poilane 12674* (holotype: P [barcode P02143106!]; isotypes: P [barcode P02143107!, P02143108!]). Image of holotype available from https://plants.jstor.org/stable/10.5555/al.ap.specimen.p02143106.

##### Distribution.

Vietnam.

#### ﻿20. Photinia prionophylla

Taxon classificationPlantaeRosalesRosaceae

(Franch.) C.K.Schneid., Repert. Spec. Nov. Regni Veg. 3: 153. 1906.

5AE45BBB-68F0-5B60-883B-98C1B3B9AB6C

≡ Eriobotrya prionophylla Franch. Pl. Delavay. 225, pl. 46. 1890. ≡ Pyrusprionophylla (Franch.) M.F.Fay & Christenh., Global Fl. 4: 116. 2018.

##### Type.

China. Yunnan: les taillis à Kiao che tong au dessus de Kiang yn, 30 May 1888, *J.M. Delavay 3545* (lectotype, designated by Idrees et al. (2021: 167): P [barcode P03342590!]; isolectotypes: K [barcode K000758254!], LE [barcode LE01015176!]). ibidem, 28 October 1888, *J.M. Delavay 3545* (syntypes: K [barcode K000758255!]). Mo-so-yn, Lau Kong, 1 June 1884, *J.M. Delavay 1077* (syntypes: A [barcode 00026479!, 00026749!, 00026750!], P [barcode P02143153!, P02143154!, P02143155!]). Image of lectotype available from https://plants.jstor.org/stable/10.5555/al.ap.specimen.k000758254.

##### Distribution.

China (Sichuan and Yunnan).

#### ﻿20a. Photinia prionophylla var. prionophylla

Taxon classificationPlantaeRosalesRosaceae

8AE838C9-78F0-507E-906D-97708CCB5DD7

##### Distribution.

China (Sichuan and Yunnan).

#### ﻿20b. Photinia prionophylla var. nudifolia

Taxon classificationPlantaeRosalesRosaceae

Hand.-Mazz., Symb. Sin. 7(3): 480. 1933.

51FD56D6-7DDB-59D9-A888-50B04CDA9C01

##### Type.

China. Yunnan: Yunnanfu (= Kunming), Prope vicum Hsiao-Magai ad septentr. urbis Yünnanfu, 25°26’ lat., in regionis calide temperatae inte Döge et Hsiaodjiadsum. 1800 m. 8 March 1914, *H. Handel-Mazzetti 404* (holotype: WU [barcode 0059448!]).

##### Distribution.

China (Yunnan).

#### ﻿21. Photinia prunifolia

Taxon classificationPlantaeRosalesRosaceae

(Hook. & Arn.) Lindl., Edwards’s Bot. Reg. 23: sub t. 1956. 1837.

83B61798-D711-5BBB-ACBB-EBFA342C0888

**Figure 9.**
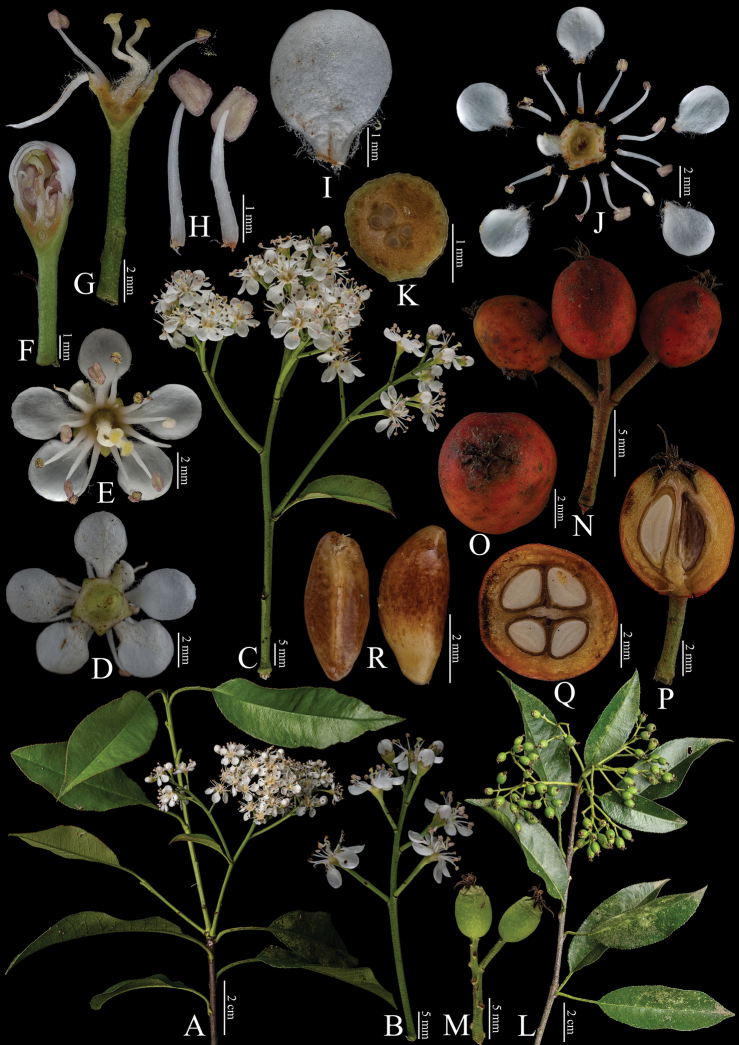
Fine structure of *Photiniaprunifolia*, encompassing various developmental stages and perspectives. **A** inflorescence branch **B, C** inflorescence branchlet **D** bottom perspective of an individual flower **E** top view of a single flower **F, G** longitudinal section through the ovary **H** stamens **I** petals **J** dissected flower showing internal structures **K** cross-section of the immature ovary **L** an infructescence branch **M, N** comparative fruit in both inmature and mature states **O** fruit, viewed from above **P** longitudinal section of fruit **Q** cross-sections of fruit **R** fully matured seed. The inflorescence branches (**A–K**) were collected on April 13, 2024. The infructescence branches in the immature state (**L, M**) were collected on October 7, 2023, while the remaining samples (**N–R**) were gathered on November 29, 2023. Ting Wang was responsible for the collection of all fresh specimens at the Hangzhou Botanical Garden (Zhejiang, China). Furthermore, Bin-Jie Ge (Chenshan Botanical Garden, Shanghai, China) dissected and photographed all the samples.

Fig. 9 Common name: 桃叶石楠 (Chinese name); pinyin (spelled as sounds in Chinese): tao ye shi nan

≡ Photinia serrulata var. prunifolia Hook. & Arn., Bot. Beechey Voy. 4: 185. 1833. ≡ Pyrusuranus M.F.Fay & Christenh., Global Fl. 4: 124. 2018.
= Photinia melanostigma Hance, J. Bot. 20: 5. 1882. Type: CHINA. Guangdong, North River, March 1881, *B.C. Henry 21691* (holotype: BM [barcode BM000602202!]). Image of holotype available from https://plants.jstor.org/stable/10.5555/al.ap.specimen.bm000602202.
= Photinia consimilis Hand.-Mazz., Anz. Akad. Wiss. Wien, Math.-Naturwiss. Kl. 59: 103. 1922. Type: CHINA. Hunan: Dschaoschan (=Shaoshan), 27 October 1917, *Handel-Mazzetti 11382* (**lectotype, designated here**: WU [barcode 0059452!]). Hunan: Shaoshan, 27 October 1917, *Handel-Mazzetti 11382* (syntype: WU [barcode 0059467!]). ibidem, 16 February 1918, *Handel-Mazzetti 11472* (syntype: WU [barcode 0059453!]). Image of lectotype available from https://plants.jstor.org/stable/10.5555/al.ap.specimen.wu0059467.
= Photinia prunifolia var. denticulata T.T.Yu, Acta Phytotax. Sin. 8(3): 228. 1963. Type: CHINA. Zhejiang, Pingyang, 28 June 1959, *S.R. Zhang 5867* (holotype: PE [barcode 00026328!]; isotypes: KUN [barcode 607582!], HTC [barcode 0003151!]).
= Photinia stapfii Chun, nom. nud.

##### Type.

China. Macao and adjacent islands, *Beechey s.n.* (lectotype, designated by Wang et al. (2019: 68): K [barcode K000758258!]; isolectotypes: E [barcode E00369054!]). Image of lectotype available from https://plants.jstor.org/stable/10.5555/al.ap.specimen.k000758258.

##### Distribution.

Cambodia, China (Fujian, Guangdong, Guangxi, Hainan, Hongkong, Hunan, Jiangxi, Zhejiang), and Vietnam.

#### ﻿22. Photinia raupingensis

Taxon classificationPlantaeRosalesRosaceae

K.C.Kuan, Acta Phytotax. Sin. 8(3): 228. 1963.

6BD25C83-2B6C-5172-9FFA-98FCEF46F3B7

≡ Pyrus raupingensis (K.C.Kuan) M.F.Fay & Christenh., Global Fl. 4: 118. 2018.

##### Type.

China. Guangdong, Raoping, Fenghuangshan, in silvis, 16 April 1931, *N.K. Chun 42691* (holotype: IBSC [barcode 0318920!]; isotypes: AU [barcode 039768!], IBK [barcode IBK00062558!, IBK00062559!], NAS [barcode NAS00374075!], PE [barcode 00020609!, 00004599!]).

##### Distribution.

China (Guangdong and Guangxi).

#### ﻿23. Photinia serratifolia

Taxon classificationPlantaeRosalesRosaceae

(Desf.) Kalkman, Blumea 21(2): 424. 1973.

205FAAA8-DF37-574D-9701-BA7FE359F0D7

≡ Crataegus serratifolia Desf., Tabl. École Bot., ed. 3 (Cat. Pl. Horti Paris.) 408. 1829. ≡ Pyrusserratifolia (Desf.) M.F.Fay & Christenh., Global Fl. 4: 121. 2018.

##### Type.

not designated.

#### ﻿23a. Photinia serratifolia var. serratifolia

Taxon classificationPlantaeRosalesRosaceae

615E40EF-6B78-5689-B839-F9EB5E78AFB3

Fig. 2C Common name: 石楠 (原变种) (Chinese name)

= Photinia glabra var. chinensis Maxim., Bull. Acad. Imp. Sci. Saint-Petersbourg, sér. 3 19(2): 179. 1873. Type: CHINA. *R. Fortune A-30* (**lectotype, designated here**: P [barcode P00781062!]; isolectotypes: P [barcode P00781061!, P00781063!, P00781064!]). Note C. Image of lectotype available from https://plants.jstor.org/stable/10.5555/al.ap.specimen.p00781062.
= Stranvaesia argyi H.Lév., Mem. Acad. Sci. Art. Barcelona ser. 3 12: 560. 1916. Type: CHINA. *Argy s.n.* (holotype: E [barcode E00011323!]). Image of holotype available from https://plants.jstor.org/stable/10.5555/al.ap.specimen.e00011323.
= Photinia serrulata var. aculeata G.H.M.Lawr., Gentes Herbarum 8: 80. 1949. Type: CHINA. Taiwan: Seisiu, *E.H. Wilson 11061* (**lectotype, designated here**: US [barcode 00097504!]; isolectotype: A [barcode 00045608!]). Image of lectotype available from https://plants.jstor.org/stable/10.5555/al.ap.specimen.us00097504.

##### Distribution.

China (Anhui, Fujian, Gansu, Guangdong, Guangxi, Guizhou, Hebei, Hubei, Hunan, Jiangsu, Jiangxi, Shaanxi, Sichuan, Taiwan, Yunnan, and Zhejiang), Indonesia, India, Japan, and Philippines.

##### Note C.

In the protologue, the author cited only one collection of specimen, *R. Fortune A-30*, four sheets of this collection have been observed in P, one preserved well (barcode P [barcode P00781062]) was designated as lectotype here.

#### ﻿23b. Photinia serratifolia var. ardisiifolia

Taxon classificationPlantaeRosalesRosaceae

(Hayata) H.Ohashi, J. Jap. Bot. 63(7): 234. 1988.

CF5F28C7-04DA-5D6C-ACFE-E4C04C666FB2

≡ Photinia ardisiifolia Hayata, Icon. Pl. Formosan. 5: 65. 1915. ≡ Photiniaserrulataf.ardisiifolia (Hayata) H.L.Li, Lloydia 14(4): 234. 1951. ≡ Photiniaserrulatavar.ardisiifolia (Hayata) K.C.Kuan, Fl. Reipubl. Popularis Sin. 36: 224. 1974.

##### Type.

China. Taiwan: Taidong, Taito, Manchosha, 1 October 1906, *G. Nakahara s.n.* (**lectotype, designated here**: TAIF [accession no. 22366!]; isolectotype: IBSC [barcode 0285883!]).

##### Distribution.

China (Taiwan).

#### ﻿23c. Photinia serratifolia var. daphniphylloides

Taxon classificationPlantaeRosalesRosaceae

(Hayata) L.T.Lu, Acta Phytotax. Sin. 38(3): 277. 2000.

BCE2FCC3-7E60-596C-884E-6A32B8EC24CE

≡ Photinia daphniphylloides Hayata, Icon. Pl. Formosan. 7: 30. 1918. ≡ Photiniaserrulataf.daphniphylloides (Hayata) H.L.Li, Lloydia 14(4): 234. 1951. ≡ Photiniaserrulatavar.daphniphylloides (Hayata) K.C.Kuan, Fl. Reipubl. Popularis Sin. 36: 222. 1974.

##### Type.

China. Taiwan: Hualian, Tarako, Batagan-sya, 27 April 1917, *S. Sasaki s.n.* (**lectotype, designated here**: TAIF [accession no. 11810!]; isolectotype: TAIF [accession no. 11811!]).

##### Distribution.

China (Taiwan).

#### ﻿23d. Photinia serratifolia var. lasiopetala

Taxon classificationPlantaeRosalesRosaceae

(Hayata) H.Ohashi, J. Jap. Bot. 63(7): 234. 1988.

717D0402-F499-5091-B9B8-F6094A8E5D2D

≡ Photinia lasiopetala Hayata, Icon. Pl. Formosan. 6: 17. 1916. ≡ Photiniaserrulatavar.lasiopetala (Hayata) K.C.Kuan, Fl. Reipubl. Popularis Sin. 36: 222. 1974. ≡ Photiniaserratifoliavar.lasiopetala (Hayata) H.Ohashi, J. Jap. Bot. 63(7): 234. 1988. ≡ Pyruslasiopetala (Hayata) M.F.Fay & Christenh., Global Fl. 4: 110. 2018.

##### Type.

China. Taiwan: Nantou, 1 April 1916, *B. Hayata s.n.* (holotype: TAIF [accession no. 11814!]; isotype: PH [barcode PH00067378!]).

##### Distribution.

China (Taiwan).

#### ﻿24. Photinia stenophylla

Taxon classificationPlantaeRosalesRosaceae

Hand.-Mazz., Symb. Sin. Pt. 7(3): 480, pl. 15, f.3. 1933.

F4BCD69A-88F3-53F8-9781-6EFB02EC7262

≡ Pyrus stenophylla (Hand.-Mazz.) M.F.Fay & Christenh., Global Fl. 4: 122. 2018.

##### Type.

China. Guizhou, Sandjio, *H. Handel-Mazzetti 10827* (lectotype, designated by Pathak et al. (2021: 41): WU [barcode 0059446!]). Sanhoa (= Sandu), Yao-ren-shan, *Y. Tsiang 6374* (syntypes: A [barcode 00026800!], NY [barcode 00436117!]). Image of lectotype available from https://plants.jstor.org/stable/10.5555/al.ap.specimen.wu0059446.

##### Distribution.

China (Guangxi and Guizhou).

#### ﻿25. Photinia taishunensis

Taxon classificationPlantaeRosalesRosaceae

G.H.Xia, L.H.Lou & S.H.Jin, Nordic J. Bot. 30(4): 439. 2012.

B820D236-CF5B-584C-BED6-EEDD36A06024

##### Type.

China. Zhejiang: Taishun County, Yangxi Village, *C.S. Ding 4116* (holotype: ZJFC [barcode 00030313!]; isotype: ZJFC [barcode 00030312!]).

##### Distribution.

China (Zhejiang).

#### ﻿26. Photinia tushanensis

Taxon classificationPlantaeRosalesRosaceae

T.T.Yu, Acta Phytotax. Sin. 8(3): 229. 1963.

CEC8F016-118E-5AC2-A2A8-959F8D69D063

≡ Pyrus tushanensis (T.T.Yu) M.F.Fay & Christenh., Global Fl. 4: 124. 2018.

##### Type.

China. Guizhou, Dushan, *Libo Exped. 1296* (holotype: PE [barcode 00020611!]; isotype: PE [barcode 01498407!]).

##### Distribution.

China (Guangxi and Guizhou).

#### ﻿27. Photinia wardii

Taxon classificationPlantaeRosalesRosaceae

C.E.C.Fisch., Bull. Misc. Inform. Kew 1936(4): 281. 1936.

54484774-F5AA-5EC9-B136-4489B7DAF70C

##### Type.

India. Assam, Chibaon, Delei Valley, *F.K. Ward 8042* (holotype: K [barcode K000758348!]; isotypes: K [barcode K000758349!, K000758350!]). Image of holotype available from https://plants.jstor.org/stable/10.5555/al.ap.specimen.k000758348.

##### Distribution.

India (Assam).

#### ﻿ Stranvaesia

Taxon classificationPlantaeRosalesRosaceae

Lindl., Edwards’s Botanical Register 23: t. 1956. 1837.

71469921-BBFD-53A3-9B9C-1C6BBC3F4E48

##### Type.

Lectotype, designated by Liu et al. (2019: 686): *Crataegusglauca* Wall. ex G.Don (= *Stranvaesianussia* (Buch.-Ham. ex D.Don) Decne.).

#### ﻿1. Stranvaesia nussia

Taxon classificationPlantaeRosalesRosaceae

(Buch.-Ham. ex D.Don) Decne., Nouv. Arch. Mus. Hist. Nat. 10: 178. 1874.

465C94EC-B72B-5D89-B54E-41116FE5F77F

≡ Pyrus nussia Buch.-Ham. ex D.Don, Prodr. Fl. Nepal. 237. 1825. ≡ Photinianussia (Buch.-Ham. ex D.Don) Kalkman, Blumea 21(2): 429. 1973.
= Crataegus glauca Wall. ex G.Don, Gen. Hist. 2: 598, descr. 1832. Type: Nepalia & Kumaon. 1829, *Wallich 673* (**lectotype, designated here**: K [barcode K000758343!, excluding the infructescence]; isolectotypes: G [barcode G00437202!, excluding the infructescence, G00437203!], GZU [barcode GZU000283039!], K [barcode K000758344!, K001111566!], L [barcode L0019509!], LE [barcode LE00013505!], M [barcode M-0213867!, M-0213868!, M-0213872!], P [barcode P02143111!], PH [barcode PH00028193!]). Image of lectotype available from https://plants.jstor.org/stable/10.5555/al.ap.specimen.k000758343.
= Stranvaesia glaucescens Lindl., Edwards’s Bot. Reg. 23: t. 1956. 1837. nom. superfl.
= Eriobotrya ambigua Merr., Publ. Bur. Sci. Gov. Lab. 35: 19. 1906. ≡ Stranvaesiaambigua (Merr.) Nakai, J. Arnold Arbor. 5: 72. 1924. Type: PHILIPPINES. Lamao River, Mt. Mariveles, Province of Bataan, Luzon, March 1905, *R. Meyer 2796* (lectotype, designated by Kalkman (1973: 429) ‘holotype’: K [barcode K000758366!]; isolectotypes: NY [barcode 00436214!], US [barcode 00097488!]). ibidem, March 1905, *H.N. Whitford 1155* (syntype: K [barcode K000758368!]). ibidem, March 1905, *H.N. Whitford 1168* (syntype: K [barcode K000758367!]). ibidem, June 1905, *H.N. Whitford 1307* (syntype: K [barcode K000758365!]). Image of lectotype available from https://plants.jstor.org/stable/10.5555/al.ap.specimen.k000758366.
= Eriobotrya oblongifolia Merr. & Rolfe, Philipp. J. Sci., C 3: 102. 1908. ≡ Rhaphiolepisoblongifolia (Merr. & Rolfe) B.B.Liu & J.Wen, Frontiers Pl. Sci. (Online journal) 10-1731: 11. 2020. Type: PHILIPPINES. Mindanao. Misamis: Mount Malindang, May 1906, *E.A. Mearns & W.J. Hutchinson 4680* (lectotype, designated by Liu et al. (2020b: 108): NY [barcode 00436215!]; isolectotype: US [barcode 00097490!]). Image of lectotype available from https://plants.jstor.org/stable/10.5555/al.ap.specimen.ny00436215.
= Photinia harmandii Cardot, Notul. Syst. (Paris) 3: 375. 1918. ≡ Stranvaesiaharmandii (Cardot) Vidal, Notul. Syst. (Paris) 13: 301. 1948. Type: LAOS. Attopeu, 1877, *Harmand 1366* (**lectotype, designated here**: P [barcode P02143112!]; isolectotype: P [barcode P02143113!]). Image of lectotype available from https://plants.jstor.org/stable/10.5555/al.ap.specimen.p02143112.

##### Type.

Nepal. Nilcunt [Nilkantha, Shading District, Bagmati Zone, Madhyamanchal, Nepal; coordinates 27.91/84.94]. *Francis Buchanan-Hamilton s.n.* (lectotype, selected by Vidal (1965: 231), first step; second step, designated by Guo et al. (2020: 110): BM [barcode BM000522002!]). *Wallich 658* (syntype: L [barcode L0062739!, L0062740!], M [barcode M-0213869!]). *Wallich 658a* (syntype: M [barcode M-0210542!]). Image of lectotype available from https://plants.jstor.org/stable/10.5555/al.ap.specimen.bm000522002.

##### Distribution.

China (Tibet and Yunnan), India, Laos, Myanmar, Nepal, Philippines, and Thailand.

#### ﻿1a. Stranvaesia nussia var. nussia

Taxon classificationPlantaeRosalesRosaceae

2DE8F8C9-9235-5D3A-917D-C6381DA57C05

##### Distribution.

China (Tibet and Yunnan), India, Laos, Myanmar, Nepal, Philippines, and Thailand.

#### ﻿1b. Stranvaesia nussia var. angustifolia

Taxon classificationPlantaeRosalesRosaceae

(Decne.) C.K.Schneid., Ill. Handb. Laubholzk. 1: 713. 1906.

09D116E4-013A-5AE7-BBA4-F4091FDC280C

≡ Stranvaesia glaucescens var. angustifolia Decne., Nouv. Arch. Mus. Hist. Nat. 10: 178. 1874.

##### Distribution.

India (Mt. Khasia).

#### ﻿2. Stranvaesia oblanceolata

Taxon classificationPlantaeRosalesRosaceae

(Rehder & E.H.Wilson) Stapf, Bot. Mag. 149: sub t. 9008. 1924.

07B0C291-E54C-5A65-AC92-86160B973ABE

≡ Stranvaesia nussia var. oblanceolata Rehder & E.H.Wilson, Pl. Wilson. (Sargent) 1: 193. 1913. ≡ Pyrusoblanceolata (Rehder & E.H.Wilson) M.F.Fay & Christenh., Global Fl. 4: 114. 2018.

##### Type.

China. Yunnan: forests around Szemao (Simao), alt. 1500–1600 m, *A. Henry 11615* (lectotype, selected by Vidal (1965: 232), first step; second step, designated by Guo et al. (2020: 110): US [barcode 00097547!]; isolectotype: A [barcode 00038562!]). ibidem, *A. Henry 11615a* (syntype: A [barcode 00038566!], K [barcode K000758307!], PE [barcode 01432740!]). ibidem, *A. Henry 11615b* (syntype: A [barcode 00038563!], K [barcode K000758306!], PE [barcode 01432741!], US [barcode 00429887!]). ibidem, *A. Henry 11615e* (syntype: A [barcode 00038564!], K [barcode K000758308!], PE [barcode 01432742!], US [barcode 00429888!]). ibidem, *A. Henry 11615f* (syntype: A [barcode 00038565!], K [barcode K000758306!]). Image of lectotype available from https://plants.jstor.org/stable/10.5555/al.ap.specimen.a00038562

##### Distribution.

China (Yunnan), Laos, Myanmar, and Thailand.

#### ﻿3. Stranvaesia lasiogyna

Taxon classificationPlantaeRosalesRosaceae

(Franch.) B.B.Liu, Molec. Phylogen. Evol. 189-107914: 11. 2023.

E9EB76BE-9B53-56D4-B9CA-23A0049C490C

≡ Eriobotrya lasiogyna Franch., Pl. Delavay. 225. 1890. ≡ Photinialasiogyna (Franch.) C.K.Schneid., Repert. Spec. Nov. Regni Veg. 3: 153. 1906. ≡ Pyrusavalon M.F.Fay & Christenh., Global Fl. 4: 96. 2018. replacement name.
= Stranvaesia glaucescens var. yunnanensis Franch., Pl. Delavay. 226. 1890. Type: CHINA. Yunnan, in silvis supra Che-tong, prope Tapin-tze, May 18, 1885, *J.M. Delavay 1992* (lectotype, designated by Idrees and Shaw (2022: 31): P barcode P02143161!; isolectotype: P barcode P02143140!). Image of lectotype available from https://plants.jstor.org/stable/10.5555/al.ap.specimen.p02143161.
= Photinia mairei H.Lév., Bull. Acad. Int. Géogr. Bot. 17: 28. 1916. Type: CHINA. rochers-brousse des mont a Kiao-me-ti, May 1911–1913, *E.E. Maire s.n.* (lectotype, designated by Pathak et al. (2021: 41): E [barcode E00011316!]; isotype: A [barcode 00038571!]). Image of lectotype available from https://plants.jstor.org/stable/10.5555/al.ap.specimen.e00011316.

##### Type.

China. Yunnan, in silvis montanis ad fauces San-tchang-kiou supra Hokin, alt. 2300 m., 22 May 1884, *J.M. Delavay 732* (lectotype, designated by Pathak et al. (2021: 40): P [barcode P02143141!]; isolectotypes: P [barcode P02143142!], US [barcode 00097489!], image A [barcode 00026747! with plant material sampled from P02143141!]). Image of lectotype available from https://plants.jstor.org/stable/10.5555/al.ap.specimen.p02143141.

##### Distribution.

China (Fujian, Guangdong, Guangxi, Hunan, Jiangxi, Sichuan, Yunnan, and Zhejiang).

#### ﻿3a. Stranvaesia lasiogyna var. lasiogyna

Taxon classificationPlantaeRosalesRosaceae

0A3609C1-54EE-5576-804E-F9D50CC848D0

Fig. 2D Common name: 倒卵叶红果树(原变种)(Chinese name)

##### Distribution.

China (Sichuan and Yunnan).

#### ﻿3b. Stranvaesia lasiogyna var. glabrescens

Taxon classificationPlantaeRosalesRosaceae

(L.T.Lu & C.L.Li) B.B.Liu, Molec. Phylogen. Evol. 189-107914: 11. 2023.

04D4A52B-808F-5744-8E04-EB1D85E88BCB

≡ Photinia lasiogyna var. glabrescens L.T.Lu & C.L.Li, Acta Phytotax. Sin. 38(3): 278. 2000.

##### Type.

China. Jiangxi, Shangrao, 4 May 1972, *Jiangxi Exped. 1071* (holotype: PE [barcode 00336583!]; isotype: PE [barcode 00336582!]).

##### Distribution.

China (Fujian, Guangdong, Guangxi, Hunan, Jiangxi, Sichuan, Yunnan, and Zhejiang).

#### ﻿ Weniomeles

Taxon classificationPlantaeRosalesRosaceae

B.B.Liu, Molec. Phylogen. Evol. 189-107914: 11. 2023.

255A2060-72C9-5A61-86C2-FAD39CA6138E

##### Type.

*Weniomelesbodinieri* (H.Lév.) B.B.Liu ≡ *Photiniabodinieri* H.Lév.

##### Distribution.

China (Anhui, Fujian, Guangdong, Guangxi, Guizhou, Hubei, Hunan, Jiangsu, Shaanxi, Sichuan, Yunnan, and Zhejiang), Indonesia, and Vietnam.

#### ﻿1. Weniomeles bodinieri

Taxon classificationPlantaeRosalesRosaceae

(H.Lév.) B.B.Liu, Molec. Phylogen. Evol. 189-107914: 12. 2023.

D604609D-7AE3-56C9-A9CB-E8503CEB9904

≡ Photinia bodinieri H.Lév., Repert. Spec. Nov. Regni Veg. 4: 334. 1907. ≡ Pyruseureka M.F.Fay & Christenh., Global Fl. 4:103. 2018. replacement name. ≡ Stranvaesiabodinieri (H.Lév.) B.B.Liu & J.Wen, J. Syst. Evol. 57(6): 686. 2019. ≡ Stranvaesiabodinieri (H.Lév.) Long Y.Wang, W.B.Liao & W.Guo, Phytotaxa 447(2): 110. 2020. later homonym.
= Photinia davidsoniae Rehder & E.H.Wilson, Pl. Wilson. 1: 185. 1912. ≡ Pyrusdavidsoniae (Rehder & E.H.Wilson) M.F.Fay & Christenh., Global Fl. 4:101. 2018. Type: CHINA, Western Hupeh (Hubei): near Ichang (Yichang), alt. 300–600 m., April 1907, *E.H. Wilson 685* (lectotype, selected by Vidal (1968), first step “type”; second step, designated by Liu et al. (2019: 687): A [barcode 00038567!] excluding the fruits and seeds in the packet; isolectotypes: BM [barcode BM000602130!], E [barcode E00011306! excluding the fruiting branch), GH [barcode 00045598! excluding the fruiting branch], HBG [barcode HBG511078! excluding the fruiting branch], US [barcode 00097494! excluding the fruiting branch]). ibidem, *E.H. Wilson 685* (paratype: A [barcode 00038567, only the fruits and seeds in the packet, 00045599!], E [barcode E00011306, excl. the flowering branch!], GH [barcode 00045598, excl. the flowering branch!], HBG [barcode HBG511078, excl. the flowering branch!], US [barcode 00097494, excl. the flowering branch!]). CHINA, Hubei: south‐west of Ichang, alt. 300 m, November 1907, *E.H. Wilson 484* (paratypes: BM [barcode BM000946991!], HBG [barcode HBG511080!]). mountains south of Ichang, May 1900, *E.H. Wilson 462* (paratypes: HBG [barcode HBG511079!], P [barcode P02143162!]). Image of lectotype available from https://plants.jstor.org/stable/10.5555/al.ap.specimen.a00038567.
= Hiptage esquirolii H.Lév., Repert. Spec. Nov. Regni Veg. 10:372. 1912. Type: CHINA, Kouy‐Tchéou (now as Guizhou): Choui‐Teou, route de Tin‐Pan‐Lo‐Fou, alt. 900 m, 4 May 1900, *J Esquirol 2097* (lectotype, designated by Liu et al. (2019: 687): E [barcode E00011307!]; isolectotypes: A [barcode 00015103!, 00045102!]). Image of lectotype available from https://plants.jstor.org/stable/10.5555/al.ap.specimen.e00011307.

##### Type.

China, Kouy-Tchéou (now Guizhou): environs de Kouy-Yang, mont. du Collège, ca et là autour des villages, 18 May 1898, *E. Bodinier 2256* (lectotype, designated by Liu et al. (2019: 686): P [barcode P02143207!]; isolectotypes: A [barcode 00045584!], E [barcode E00010998!], P [barcode P02143208!, P02143209!]). Image of lectotype available from https://plants.jstor.org/stable/10.5555/al.ap.specimen.p02143207.

##### Distribution.

China (Anhui, Fujian, Guangdong, Guangxi, Guizhou, Hubei, Hunan, Jiangsu, Shaanxi, Sichuan, Yunnan, and Zhejiang), Indonesia, and Vietnam.

#### ﻿1a. Weniomeles bodinieri var. bodinieri

Taxon classificationPlantaeRosalesRosaceae

C8EB88E0-A0A8-5932-A636-08CD5247CDC3

Fig. 2E , 10 Common name: 椤木(原变种)(Chinese name)

##### Distribution.

China (Anhui, Fujian, Guangdong, Guangxi, Guizhou, Hubei, Hunan, Jiangsu, Shaanxi, Sichuan, Yunnan, and Zhejiang), Indonesia, and Vietnam.

**Figure 10.**
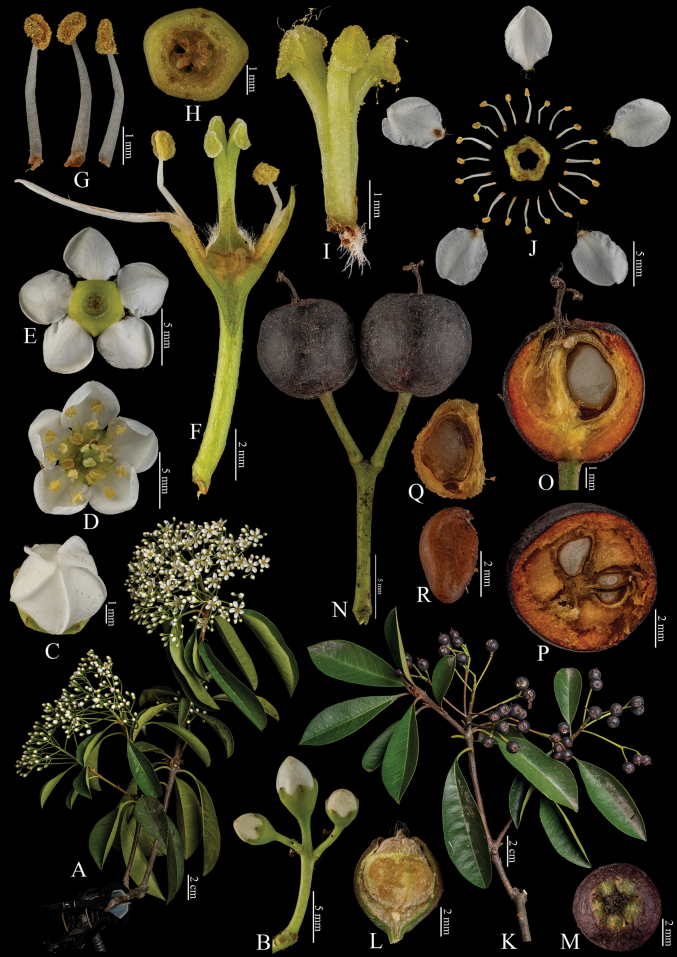
Comprehensive structural anatomy of *Weniomelesbodinieri*. **A** branch of the inflorescence **B** flowers **C, D** comparative top view of a single flower in both unopened and opened states **E** bottom perspective of an individual flower **F** longitudinal section through the ovary **G** stamens **H** cross-section through the ovary **I** detailed view of the pistil **J** dissected flower, illustrating internal structure **K** branch of the infructescence **L** cross-section of a young fruit **M** top view of a developing fruit **N** mature fruits **O** longitudinal-section through a mature fruit **P** cross-section through a mature fruit **Q** cross-section through a mature seed **R** a fully matured seed. The inflorescence branches were collected on June 14, 2022, and the infructescence branches were gathered on December 15, 2023, by Bin-Jie Ge at the Chenshan Botanical Garden, Shanghai. Additionally, Bin-Jie Ge dissected and photographed all the samples.

#### ﻿1b. Weniomeles bodinieri var. longifolia

Taxon classificationPlantaeRosalesRosaceae

(Cardot) B.B.Liu, Molec. Phylogen. Evol. 189-107914: 13. 2023.

897A758D-BEE1-5E47-A84F-3A28A70E8844

≡ Photinia bodinieri H.Lév. var. longifolia Cardot, Notul. Syst. (Paris) 3: 374. 1918. ≡ Stranvaesiabodinierivar.longifolia (Cardot) B.B.Liu & J.Wen, J. Syst. Evol. 57(6): 687. 2019.

##### Type.

China, Kouei Tchéou (now as Guizhou Province): grande route Kouei Tchéou au Kuangsi (Guangxi Province), Kout’ong (now as Gudong Xiang, Pingtang County), 22 May 1899, *Beauvais J. 175* (lectotype, designated by Liu et al. (2019: 687): P [barcode P02143211!]; isolectotype: P [barcode P02143210!]). Image of lectotype available from https://plants.jstor.org/stable/10.5555/al.ap.specimen.p02143211.

##### Distribution.

China (Guizhou).

#### ﻿1c. Weniomeles bodinieri var. ambigua

Taxon classificationPlantaeRosalesRosaceae

(Cardot) B.B.Liu, Molec. Phylogen. Evol. 189-107914: 13. 2023.

F0C3CA7F-E0AA-5920-B3DA-4D95C40EF858

≡ Photinia davidsoniae var. ambigua Cardot, Notul. Syst. (Paris) 3: 374. 1918.

##### Type.

China, Su-Tchuen (Sichuan): Eul Se Yug, vallée du Yalory, alt. 2000 m, 5 May 1911, *Legendre 834* (lectotype, designated by Jin et al. (2023: 13): P [barcode P02143164!]; isolectotype: P [barcode P02143165!). Image of lectotype available from https://plants.jstor.org/stable/10.5555/al.ap.specimen.p02143164.

##### Distribution.

China (Sichuan).

#### ﻿1d. Weniomeles bodinieri var. pungens

Taxon classificationPlantaeRosalesRosaceae

(Cardot) B.B.Liu, Molec. Phylogen. Evol. 189-107914: 13. 2023.

CB260A3D-0990-5F42-A8D1-57857E8B6669

≡ Photinia davidsoniae Rehder & E.H.Wilson var. pungens Cardot, Notul. Syst. (Paris) 3: 374. 1918.

##### Type.

China, Hubei: Ichang, *A. Henry 7174* (holotype: P [barcode P02143163!). Image of holotype available from https://plants.jstor.org/stable/10.5555/al.ap.specimen.p02143163.

##### Distribution.

China (Hubei).

#### ﻿2. Weniomeles atropurpurea

Taxon classificationPlantaeRosalesRosaceae

(P.L.Chiu ex Z.H.Chen & X.F.Jin) B.B.Liu comb. nov.

7E7788B5-06D3-585D-B3EA-57F1A6933B71

urn:lsid:ipni.org:names:77342734-1

≡ Photinia atropurpurea P.L.Chiu ex Z.H.Chen & X.F.Jin, J. Hangzhou Univ., Nat. Sci. Ed. 20(4): 393. 2021.

##### Type.

China, Zhejiang: Taishun, Zuoxi, Lishuqiu, alt. 400 m, 3 May 2020, *Z.H. Chen, Z.P. Lei & W.Y. Xie TS20050316* (holotype: ZM; isotype: ZM).

##### Distribution.

China (Zhejiang).

## ﻿Conclusion

In summary, our study addresses the long-standing deficiency of the comprehensive phylogenetic backbone in the apple tribe Maleae, primarily stemming from limited taxon and marker sampling in prior research efforts. Our phylogenomic investigations conclusively identified three major clades within the tribe. Integrating evidence from nuclear phylogeny, morphology, and ploidy estimation, we present an updated infra-tribal taxonomic system, introducing subtribe Malinae Reveal, subtribe Lindleyinae Reveal, and subtribe Vauqueliniinae B.B.Liu (subtr. nov.). Notably, our plastid phylogenetic analysis underscored the monophyly of most genera, albeit with exceptions such as *Amelanchier*, *Malus*, *Sorbus* s.l., and *Stranvaesia*. Furthermore, we contribute a comprehensive taxonomic synopsis of *Photinia* and its morphological counterparts in the Old World, recognizing and delineating 27 species along with ten varieties within *Photinia*, three species and two varieties within *Stranvaesia*, and two species paired with three varieties within *Weniomeles*. Additionally, our study makes a valuable contribution by lectotypifying 12 names and making two new combinations, thereby aiding in clarifying nomenclatural ambiguities.

## Supplementary Material

XML Treatment for Malus

XML Treatment for Malinae

XML Treatment for Lindleyinae

XML Treatment for Vauqueliniinae

XML Treatment for Photinia

XML Treatment for Photinia anlungensis

XML Treatment for Photinia beckii

XML Treatment for Photinia berberidifolia

XML Treatment for Photinia chihsiniana

XML Treatment for Photinia chingiana

XML Treatment for Photinia chingiana var. chingiana

XML Treatment for Photinia chingiana var. lipingensis

XML Treatment for Photinia chiuana

XML Treatment for Photinia crassifolia

XML Treatment for Photinia cucphuongensis

XML Treatment for Photinia davidiana

XML Treatment for Photinia davidiana var. davidiana

XML Treatment for Photinia davidiana var. undulata

XML Treatment for Photinia glabra

XML Treatment for Photinia griffithii

XML Treatment for Photinia integrifolia

XML Treatment for Photinia integrifolia var. integrifolia

XML Treatment for Photinia integrifolia var. flavidiflora

XML Treatment for Photinia integrifolia var. notoniana

XML Treatment for Photinia integrifolia var. sublanceolata

XML Treatment for Photinia lanuginosa

XML Treatment for Photinia lindleyana

XML Treatment for Photinia lindleyana var. lindleyana

XML Treatment for Photinia lindleyana var. yunnanensis

XML Treatment for Photinia lochengensis

XML Treatment for Photinia loriformis

XML Treatment for Photinia maximowiczii

XML Treatment for Photinia megaphylla

XML Treatment for Photinia microphylla

XML Treatment for Photinia prionophylla

XML Treatment for Photinia prionophylla var. prionophylla

XML Treatment for Photinia prionophylla var. nudifolia

XML Treatment for Photinia prunifolia

XML Treatment for Photinia raupingensis

XML Treatment for Photinia serratifolia

XML Treatment for Photinia serratifolia var. serratifolia

XML Treatment for Photinia serratifolia var. ardisiifolia

XML Treatment for Photinia serratifolia var. daphniphylloides

XML Treatment for Photinia serratifolia var. lasiopetala

XML Treatment for Photinia stenophylla

XML Treatment for Photinia taishunensis

XML Treatment for Photinia tushanensis

XML Treatment for Photinia wardii

XML Treatment for Stranvaesia

XML Treatment for Stranvaesia nussia

XML Treatment for Stranvaesia nussia var. nussia

XML Treatment for Stranvaesia nussia var. angustifolia

XML Treatment for Stranvaesia oblanceolata

XML Treatment for Stranvaesia lasiogyna

XML Treatment for Stranvaesia lasiogyna var. lasiogyna

XML Treatment for Stranvaesia lasiogyna var. glabrescens

XML Treatment for Weniomeles

XML Treatment for Weniomeles bodinieri

XML Treatment for Weniomeles bodinieri var. bodinieri

XML Treatment for Weniomeles bodinieri var. longifolia

XML Treatment for Weniomeles bodinieri var. ambigua

XML Treatment for Weniomeles bodinieri var. pungens

XML Treatment for Weniomeles atropurpurea

## ﻿Additional information

### Author ORCIDs

Hui Wang https://orcid.org/0009-0009-9075-698X

Xiao-Ya Li https://orcid.org/0009-0004-7164-0993

Yan Jiang https://orcid.org/0009-0004-3787-4577

Ze-Tao Jin https://orcid.org/0000-0003-1358-0043

Dai-Kun Ma https://orcid.org/0009-0005-5523-508X

Bing Liu https://orcid.org/0000-0002-6086-253X

Chao Xu https://orcid.org/0000-0002-9678-4772

Bin-Jie Ge https://orcid.org/0000-0002-4232-3567

Ting Wang https://orcid.org/0009-0007-1311-1761

Qiang Fan https://orcid.org/0000-0003-4254-6936

Shui-Hu Jin https://orcid.org/0000-0003-0334-6683

Guang-Ning Liu https://orcid.org/0009-0009-0765-0392

Bin-Bin Liu https://orcid.org/0000-0002-0297-7531

### Data availability

All of the data that support the findings of this study are available in the main text or Supplementary Information.

## Supplementary materials

Supplementary material 1 Phylogenetic tree of the apple tribe Maleae extimated through maximum likelihood analysis using IQ-TREE2 Data type pdf Explanation note Phylogenetic tree of the apple tribe Maleae extimated through maximum likelihood analysis using IQ-TREE2, based on the whole plastome dataset. The numbers displayed above each branch represent the SH-aLRT support values and Ultrafast Bootstrap support percentages, respectively. https://binary.pensoft.net/file/1060371This dataset is made available under the Open Database License (http://opendatacommons.org/licenses/odbl/1.0/). The Open Database License (ODbL) is a license agreement intended to allow users to freely share, modify, and use this Dataset while maintaining this same freedom for others, provided that the original source and author(s) are credited.Hui Wang, Xiao-Ya Li, Yan Jiang, Ze-Tao Jin, Dai-Kun Ma, Bing Liu, Chao Xu, Bin-Jie Ge, Ting Wang, Qiang Fan, Shui-Hu Jin, Guang-Ning Liu, Bin-Bin Liu

Supplementary material 2 Phylogenetic tree of the apple tribe Maleae extimated through maximum likelihood analysis using RAxML Data type pdf Explanation note Phylogenetic tree of the apple tribe Maleae extimated through maximum likelihood analysis using RAxML, based on the whole plastome dataset. The numbers displayed above each branch represent the Bootstrap support value. https://binary.pensoft.net/file/1060372This dataset is made available under the Open Database License (http://opendatacommons.org/licenses/odbl/1.0/). The Open Database License (ODbL) is a license agreement intended to allow users to freely share, modify, and use this Dataset while maintaining this same freedom for others, provided that the original source and author(s) are credited.Hui Wang, Xiao-Ya Li, Yan Jiang, Ze-Tao Jin, Dai-Kun Ma, Bing Liu, Chao Xu, Bin-Jie Ge, Ting Wang, Qiang Fan, Shui-Hu Jin, Guang-Ning Liu, Bin-Bin Liu
