# Supplementary figures and images for: ﻿Refining the phylogeny and taxonomy of the apple tribe Maleae (Rosaceae): insights from phylogenomic analyses of 563 plastomes and a taxonomic synopsis of Photinia and its allies in the Old World
Source: PhytoKeys. 2024 May 31;242:161–227. doi: 10.3897/phytokeys.242.117481 (PMC11161682; doi:10.3897/phytokeys.242.117481)

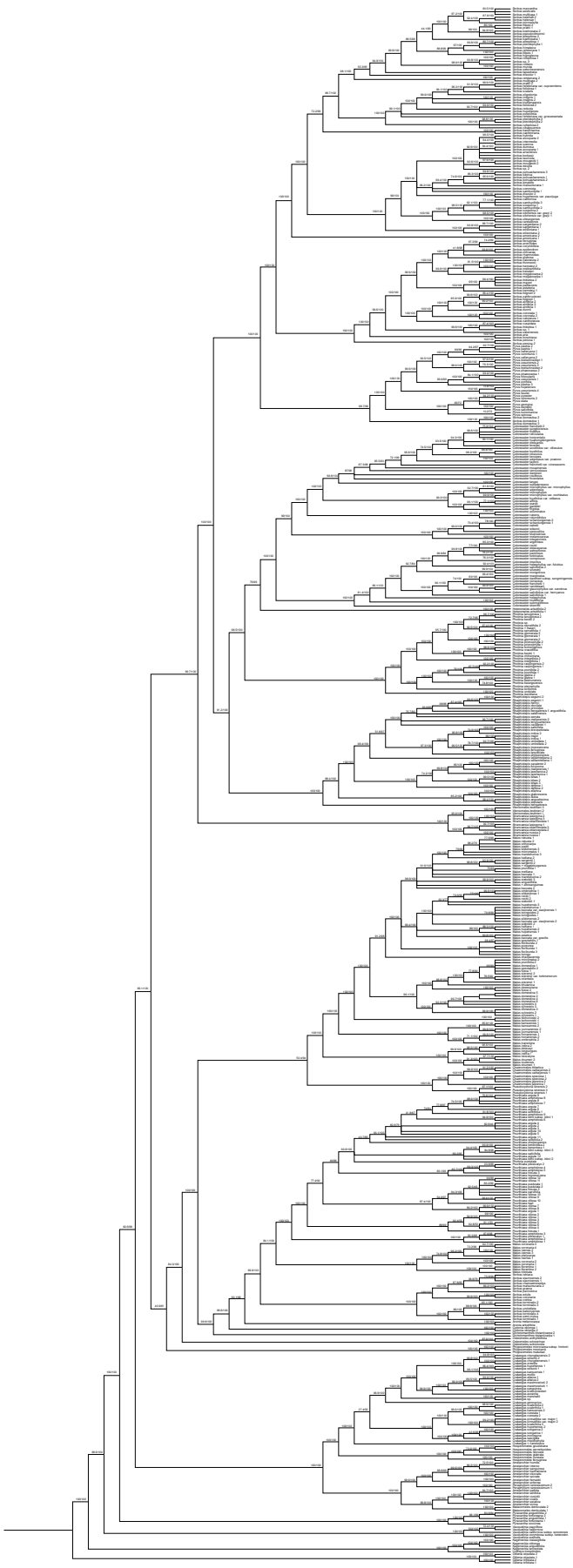

Supplement: Supplementary material 1 — Phylogenetic tree of the apple tribe Maleae extimated through maximum likelihood analysis using IQ-TREE2 [file phytokeys-242-161_article-117481__-s001.pdf]

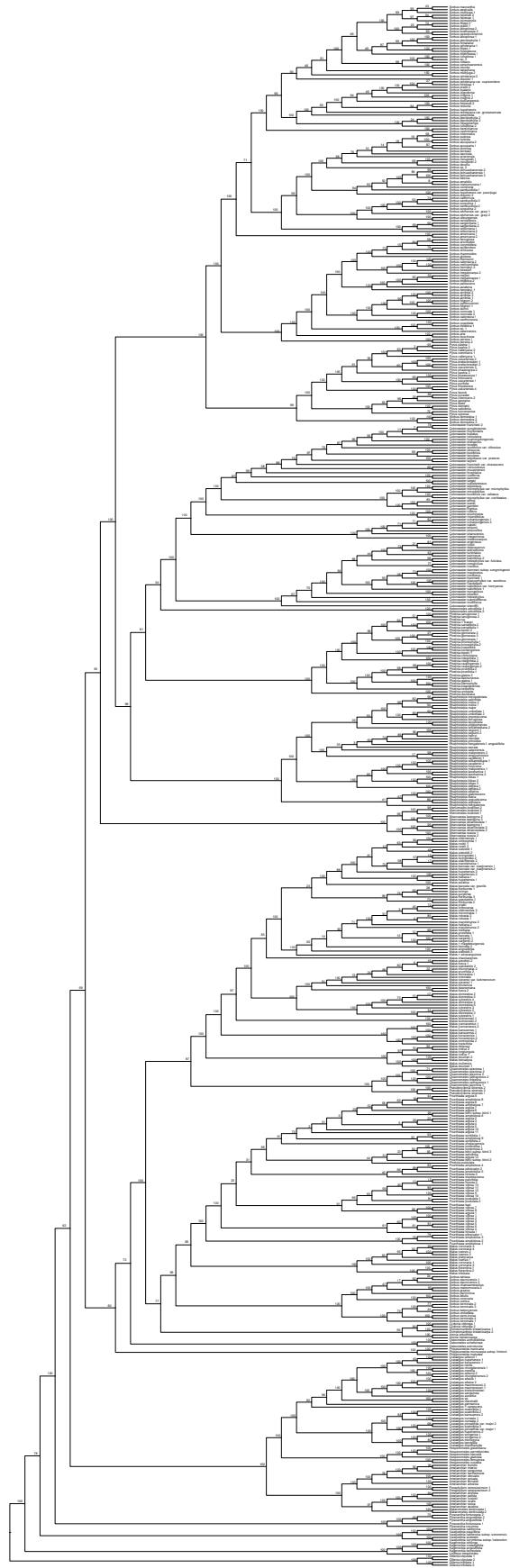

Supplement: Supplementary material 2 — Phylogenetic tree of the apple tribe Maleae extimated through maximum likelihood analysis using RAxML [file phytokeys-242-161_article-117481__-s002.pdf]
